# Does mindfulness training modulate the influence of spatial attention on the processing of intracutaneous electrical stimuli?

**DOI:** 10.1371/journal.pone.0201689

**Published:** 2018-08-09

**Authors:** Rob H. J. Van der Lubbe, Elian De Kleine, Karlein M. G. Schreurs, Ernst T. Bohlmeijer

**Affiliations:** 1 Cognitive Psychology and Ergonomics, University of Twente, Enschede, The Netherlands; 2 Laboratory of Vision Science and Optometry, Faculty of Physics, Adam Mickiewicz University, Poznań, Poland; 3 Psychology, Health & Technology, University of Twente, Enschede, The Netherlands; University of British Columbia, CANADA

## Abstract

Mindfulness based stress reduction (MBSR) training has been proposed to improve attentional skills by modulating thalamo-cortical loops that affect the sensitivity of relevant cortical areas like the somatosensory cortex. This modulation may be reflected in the electroencephalographic (EEG) alpha rhythm, and could affect the processing of subsequently applied intracutaneous electrical stimuli. Participants took part in an MBSR training and participated in two EEG sessions. EEG was measured in variants of an endogenous orienting paradigm in which attention had to be directed to the left or right forearm. After the orienting interval, the electrical stimulus was applied, equally likely on the attended or the unattended forearm. One group of participants took part in the EEG session before and after the training, while the other group took part after the training, and another time, eight weeks later. The influence of the MBSR training and spatial attention were examined with behavioral measures, lateralized alpha power within the orienting interval, and with event-related potentials (ERPs) evoked by the electrical stimuli. Self-reported mindfulness was clearly affected by the training, but no influence was found on other behavioral measures. Alpha power was clearly lateralized due to spatial attention and several ERP components (N130, N180, P340) were modulated by spatial attention but no support was found for an influence of the MBSR training. Finally, analyses revealed that individual differences in training time modulated some of the observed effects, but no support was found for an influence on attentional orienting.

## Introduction

In the last decades, several authors have argued that meditation training techniques like mindfulness based stress reduction (MBSR [1]) improve the efficiency of attentional orienting [2–5]. It has also been reported that MBSR leads to decreased pain sensations [6], which may be related to an improved ability to disengage attention from the painful area. The goal of the current study was to examine whether different attentional strategies directly affect the processing of painful stimuli, by focusing on various measures derived from the electroencephalogram (EEG), and to test whether these attentional strategies are indeed more effective after the MBSR training. For an extensive review of the literature on the influence of different types of meditation on EEG, event-related potentials (ERPs) and other neuroimaging measures, see Cahn and Polich [7].

The standard MBSR training takes eight weeks and involves meditative techniques like the body scan, sitting meditation, and yoga. During the body scan, practitioners direct their attention to many parts of their body, starting with one of the toes and ending with the top of the head. This type of training may be denoted as focused attention meditation [5]. If the ability to focus attention advances, this may result in reduced distraction and in an improved ability to monitor the content of experience, which may help to recognize the nature of emotional and cognitive patterns. The latter type is called open monitoring meditation [5]. An interesting view regarding the effectiveness of the MBSR training holds that learning to attend to one’s breathing and body sensations optimizes the filtering of inputs to sensory cortices, which in the end may also extend to distressing thoughts [2]. At the neurophysiological level, this filtering might occur by selective modulation of thalamo-cortical loops to relevant sensory areas, which is controlled by prefrontal cortical areas. This selective modulation may be reflected in local changes in the alpha (α) rhythm of the EEG [2].

Kerr et al. (2011 [8]) used the magneto-electroencephalogram (MEG) to examine whether modulation in the α band (~ 8–12 Hz) can be enhanced by the MBSR training. Participants were randomly assigned to either an MBSR or a control group. A task was used in which a cue instructed participants to attend to either the left hand, the left foot, or both. Participants had to detect whether a tactile stimulus was applied to the cued body area after a time interval varying from 1,100 to 2,100 ms. For the 600–800 ms time window after presenting the cue, a reduction in α power was observed above hand-motor areas after hand cues relative to foot cues in the MBSR group, while no such difference was observed in the control group. These findings may indicate that the MBSR training increases attentional control over activity in the somatosensory cortex before applying the tactile stimuli. Although these differences were not long-lasting as no effects were observed for the subsequent 800–1,000 ms interval, these changes might imply enhanced tactile sensitivity for the MBSR group relative to the controls.

**Fig 1:**
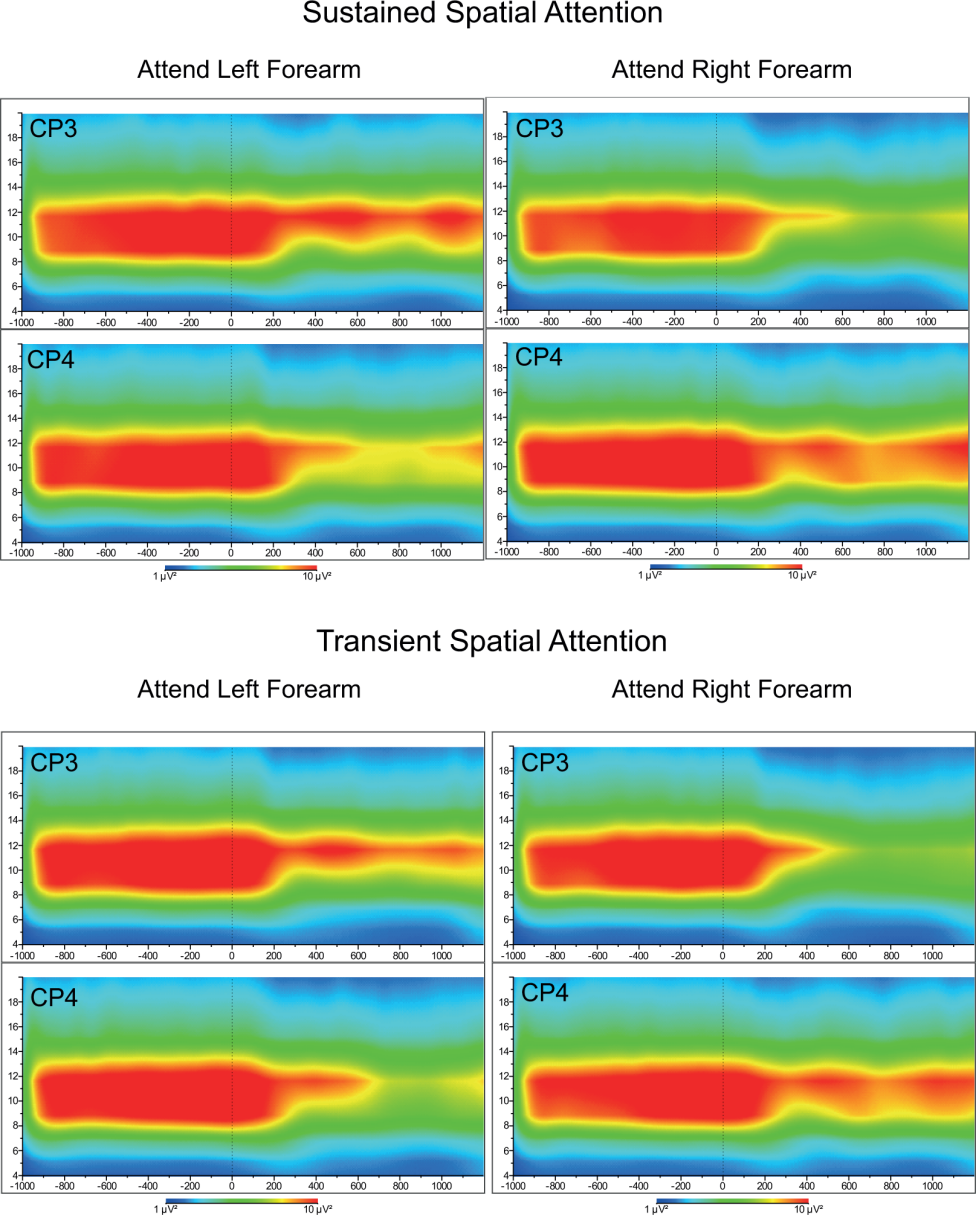
Time-frequency maps for electrodes overlaying the somatosensory areas related to the left (CP4) and the right hands (CP3). At time point “0”, a cue signaled whether the left or the right forearm had to be attended. Before cue onset, alpha (α) power (~ 8–12 Hz) is very dominant at both electrodes. Importantly, at about 400 ms after cue onset there is a clear reduction in α power over contralateral electrodes (i.e., CP4 for left hand cues, CP3 for right hand cues) and less over ipsilateral electrodes. The displayed maps are based on grand averages of the transient and sustained spatial attention conditions, described in the methods section of the current paper. In the sustained attention condition, the cued side was constant within a block of trials, while in the transient attention condition, the cued side varied from trial to trial.

Recently, Van der Lubbe et al. [9] used another paradigm that can be used to examine local changes above somatosensory areas while anticipating painful stimuli. This paradigm can be considered as a variant of the paradigm employed by Worden et al. [10] with somatosensory instead of visual target stimuli. Now, a symbolic visual cue presented on each trial informed participants whether they had to attend to their left or their right forearm. One thousand ms after the onset of the cue an intracutaneous electrical stimulus was applied to the left or the right hand. The cued side and the side of the electrical stimulus were unrelated, but participants had to press a foot pedal when the stimulus occurred on the attended side and when the intensity of this stimulus (low or high) was defined as task relevant. Van der Lubbe et al. revealed that EEG activity in the α and beta (β) band (~ 12–20 Hz) during the orienting phase, thus while participants anticipated the electrical stimuli, was selectively reduced above contralateral relative to ipsilateral somatosensory areas [9]. They used so-called lateralized power spectra (LPS; see [11]), which are normalized power differences between contra- and ipsilateral sites for a specific frequency band that correct for overall differences between participants and hemispherical differences. As this EEG measure is not that well known and quite relevant at the same time for the present paper, we clarified it on the basis of data acquired in the current study. In Fig 1, time-frequency maps are displayed for two electrodes overlaying the somatosensory areas related to the left and right hands.

A clear reduction in contralateral alpha power is visible 400 ms after the to-be-attended hand has been cued, while ipsilateral power stays relatively high for both hands. Thus, the difference between contralateral and ipsilateral power seems related to attentional orienting towards the hands and therefore may be used as an electrophysiological index of somatosensory spatial attention. In an earlier paper, Van der Lubbe and Utzerath [11] proposed to extend the normalization procedure employed by Thut et al. [12] to a double subtraction, in analogy to the computation of the lateralized readiness potential (e.g., see Coles, 1989 [13]). This extension results in an index that was called the LPS. If we apply this logic to the CP3 and CP4 electrodes, the LPS is computed as indicated in Eq 1.

$$\text{LPS}\left(\text{CP}4/3\;\omega \text{p}t\right)=\left(\left(\text{left hand cues}\frac{\left(\text{CP}3\;\omega \text{p}t\;–\;\text{CP}4\;\omega \text{p}t\right)}{\left(\text{CP}3\;\omega \text{p}t\;+\;\text{CP}4\;\omega \text{p}t\right)}-\;\left(\text{right hand cues}\frac{\left(\text{CP}4\;\omega \text{p}t\;–\;\text{CP}3\;\omega \text{p}t\right)\;}{\left(\text{CP}3\;\omega \text{p}t\;+\;\text{CP}4\;\omega \text{p}t\right)\;}\right)\right)/2\right)\;$$ (1)

A positive value of the LPS indicates that contralateral power (p) in a specific frequency band (*ω*) at a specific moment in time (*t*) is smaller than ipsilateral power, while a negative value suggests the opposite. In the study of Van der Lubbe et al. [9], positive values were observed, which indicates that power in both the α and β bands was reduced above contralateral somatosensory areas, in line with Kerr et al.’s study [8]. Activity in the α band has been viewed as an inhibitory rhythm that filters out irrelevant activity. Attentional orienting is thought to attenuate this filtering and thereby prioritize attended above unattended information (see [14–15]). The central idea with regard to the influence of the MBSR training as proposed by Kerr et al. [2] is that MBSR specifically enhances this ability to filter and prioritize information processing. One possibility, explored in the current paper, is that the MBSR training facilitates the ability to keep attention consistently focused on a relevant side for a longer time, which accords with the findings reported by Lutz et al. [3]. This may be examined with a sustained spatial attention modulation (see [9]). Another possibility, also explored here, is that the MBSR training specifically facilitates the switching of attention from side to side, which might show up in a transient spatial attention paradigm (e.g., see [16]).

As the MBSR training may affect attentional orienting it should also influence the processing of subsequently presented stimuli. The modulation of ERP components by spatial attention has been dominated by ERP effects on visual stimuli (e.g., see [17–18]; for a comprehensive overview up to 2007, see Wright & Ward [19]). Likewise, several studies revealed that specific ERP components after painful stimuli are affected by spatial attention. Blom et al. [20] examined whether sustained distraction (participants were asked to perform a word association, a mental arithmetic or an attention task) modulates ERP components evoked by electrocutaneous painful stimuli. Their results revealed an attenuation of the sensory N1 component due to distraction that peaked at about 130 ms after stimulus onset, and also a reduction of the anterior P2/P3a component that peaked around 260 ms after stimulus onset. These observations suggest that distraction leads to diminished processing in somatosensory areas, and also to a reduced orienting effect towards the painful stimuli.

Another study of Van der Lubbe et al. [16] used a transient manipulation of spatial attention, by varying the to-be-attended location from trial to trial with a centrally presented cue. Like in the study of Blom et al. the amplitudes of early negative ERP components (N100 and N150) were reduced for unattended stimuli, which on the basis of source analyses could be related to changes in the secondary somatosensory cortex. However, the later positive anterior component (P260) was now enlarged for unattended stimuli of high intensity, which was interpreted as a “call for attention”. The study of Van der Lubbe et al. [9] revealed that this effect (now on the P340, so slightly later, likely due to more selective processing along nociceptive pathways) was also present when attention was consistently directed to one side (i.e., sustained spatial attention), which led to the conclusion that task relevance of the stimuli plays a crucial role in the enlarged P340 component to unattended stimuli.

In the current study, a comparable manipulation as in the study of Kerr et al. [8] was employed to examine the influence of the MBSR training. In our study, participants were also randomly allocated to two groups but both groups were measured in two sessions, which allows to control for general group differences. The first group (the pre-post group) took part in the EEG experiment before and after the MBSR training. The second group (the post-post2 group) participated directly after the MBSR training, while the second session was carried out about eight weeks later. This setup differs from the common approach to measure one group before and after a specific intervention (e.g., MBSR training) and another group before and after a control manipulation, (e.g., rest, see [21]). Our approach has the advantage that both groups receive the same intervention, which reduces potential motivational differences between participants in both groups. The crucial difference is that the predicted effects are reversed in time, as we may expect group differences in the first session, but not in the second session, as both groups already received the intervention (for further considerations related to this design, see discussion). Two versions of a spatial attention task with intracutaneous electrical stimuli of two intensities were employed. In the sustained version, participants had to attend to the left (or the right) forearm during a series of trials, while in the transient version, the to-be-attended side varied from trial to trial. We assumed that the MBSR training will improve participants’ ability to focus and sustain their attention [3, 5], while over time the training may also lead to an increased ability to disengage attention from aversive stimuli [4]. We expected that effects of the MBSR training will be reflected in the results of a questionnaire that examined the subjective experience of mindfulness [22]. The training might also modulate pain tolerance thresholds, intensity ratings of the electrical stimuli and the common reduction of these ratings in the course of the experiment (e.g., see [9,20,23]. An increased ability to focus attention may be reflected in improved perceptual sensitivity, assessed with *d'*, a measure derived from signal detection theory [24]. Furthermore, improved sensitivity may be strongest in the sustained attention conditions (see [5]). Altogether, we expected to observe behavioral and self-report differences due to the MBSR training in the first measurement session, but not in the second measurement session.

Specific effects can be predicted regarding the influence of spatial attention and its modulation due to the MBSR training on various EEG measures. In the orienting phase, a reduction in contralateral relative to ipsilateral α power above somatosensory areas, assessed with the LPS method [11] was expected to be observed, which might extend to parietal/occipital areas. This reduction may be more pronounced and/or more focal in the case of sustained than in the case of transient spatial attention (see [9]). Most importantly, these effects were expected to be stronger in the first session for the post-post2 MBSR group than for the pre-post MBSR group due to the MBSR training, while these group differences may disappear in the second session.

The processing of the intracutaneous electrical stimuli within somatosensory areas is expected to be reflected in early negative components (N130, N180; see [9]) whose amplitudes are predicted to be larger for attended than for unattended stimuli, and larger for high intensity than for low intensity stimuli. We expected effects of attention condition (transient vs. sustained), but most importantly, group-related attentional differences in the first session but not in the second session. In our previous studies [9,16,23] we observed an enlarged late positive component for unattended high intensity stimuli (P3a or P340) that we interpreted as a “call for attention” (see [25]). This effect on the P3a might be substantially reduced in the first session for the post-post2 group as they already took part in the MBSR training and therefore may be less distracted [4], while this group difference might disappear in the second session due to stable changes in attentional control. Finally, there might be individual differences within groups as individual training times will likely differ. This possible influence will be examined in sub-analyses per group and session by including individual training time as a covariate.

## Materials and methods

### Participants

Thirty-four healthy students (age range: 20–34 years) participated in two experimental sessions (each with a duration of about three hours) in exchange for a free standardized MBSR training. All participants had normal or corrected-to-normal vision and reported to be free of neurological and psychiatric disorders. Every participant received a detailed explanation of the procedure and signed a written informed consent before participating. The Medical Ethical Committee of Medisch Spectrum Twente approved the employed experimental procedures (NL31474.044.11/P11-11). Participants were randomly assigned to one of two groups, denoted as the pre-post group and the post-post2 group. Both groups took part in an eight-week standardized MBSR training. The pre-post group completed an experimental session with EEG eight weeks before and directly after the MBSR training, while the post-post2 group completed the first session directly after the MBSR training, and the second session eight weeks later. Data of the first session for the pre-post group, so before participants took part in the MBSR training, were reported in the study of Van der Lubbe et al. [9].

None of the participants had been involved in a mindfulness training session and an experiment involving painful stimulation before. Data of six participants were excluded due to recording and procedural problems in one of the two sessions. The analyses were carried out on the data from 28 participants (25 female, 3 male, M_age_ = 23.8 years, SE = 0.6, all right-handed), 15 in the pre-post group and 13 in the post-post2 group.

### MBSR training

The eight-weeks of the MBSR training consisted of weekly group sessions of 2.5 hours and of homework assignments (five days a week, 45 minutes a day). The MBSR protocol was largely followed, but we did not include a full-day session at the end of the training. An experienced meditation and yoga teacher carried out the training. The exercises included the body-scan, mindfulness, meditation, meditative walking, yoga, and mindful-communication [26]. The aim of the training was to develop attentiveness in daily life of the participants.

### Questionnaire

The Five Facet Mindfulness Questionnaire–short form (FFMQ-SF; [22]) was administered to all participants at the start of the study, after the mindfulness training (right before the EEG measurements), and additionally in the post-post2 group at the start of the second experimental session. After filling in the questionnaire at the start of the first and the second experimental session, the EEG and stimulation electrodes were attached.

### Stimuli and task

**Fig 2:**
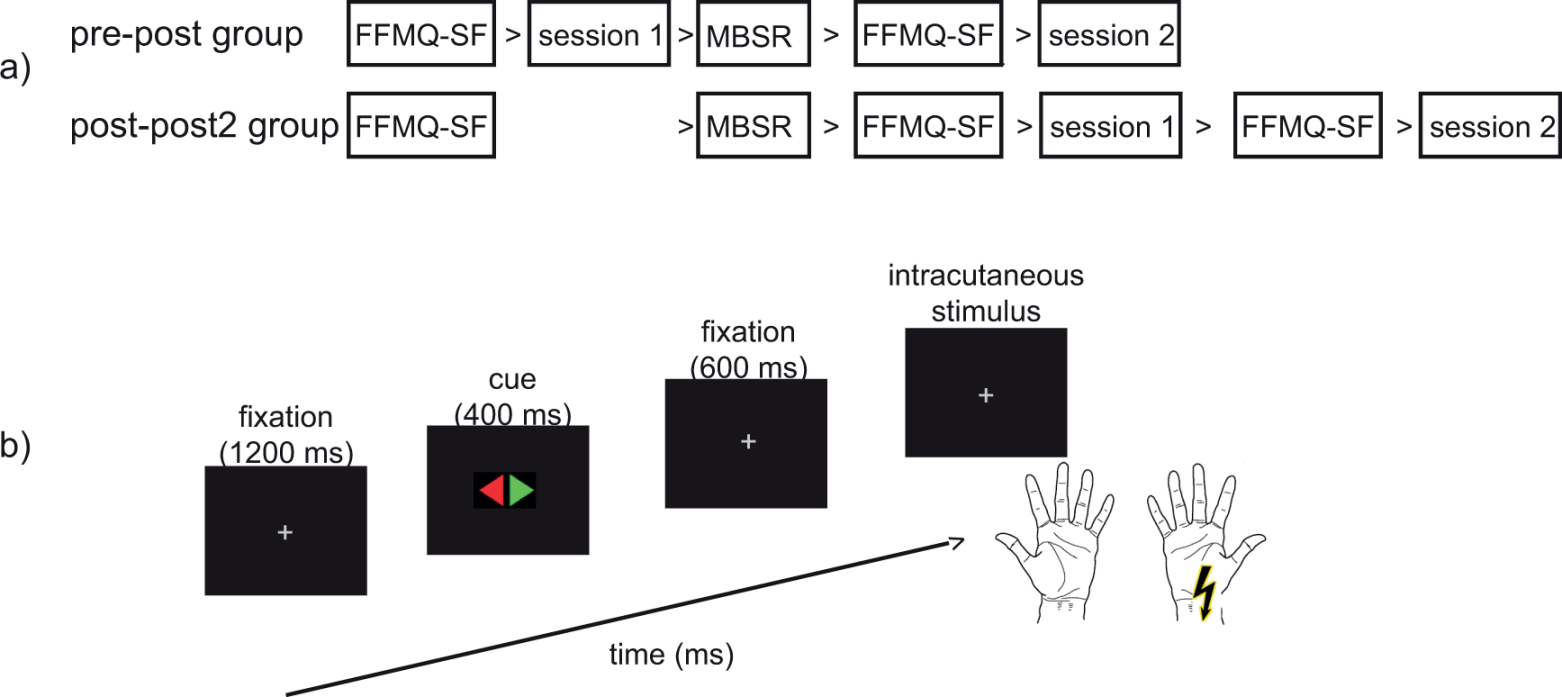
a) The employed design of the experiment. Participants were divided into two groups: the pre-post group and the post-post2 group. Both groups participated in two experimental sessions. The pre-post group took part in a session before and after the MBSR (mindfulness based stress reduction) training, while both sessions of the post-post2 group, separated by about six weeks, were carried out after the training. Self-reports of mindfulness were assessed with the FFMQ-SF (Five Facet Mindfulness Questionnaire–short form) questionnaire before the start of the experiment, and shortly before the experimental sessions. b) The sequence of events on an experimental trial; after a fixation period, a centrally presented cue signaled the to-be-attended hand. One thousand milliseconds after cue onset, an intracutaneous electrical stimulus was applied that could equally likely occur on the attended or the unattended side. A foot pedal response was only required when the stimulus occurred on the attended side and when it had the relevant (low or high) intensity, which was varied between participants.

Participants sat on a chair and were located at approximately 60 cm in front of a computer monitor. An example of the stimuli, their order, and their exposure times is depicted in Fig 2. Each trial started with a white fixation cross (0.48° * 0.48°) displayed in the center of the monitor. After 1,200 ms, the fixation cross was replaced by a rhomb (the visual cue; 3.62° * 1.91°) for a duration of 400 ms. The rhomb consisted of a red and a green triangle. One of the triangles signaled the to-be-attended side, which depended on the relevant cue color in that specific block of trials. In two successive blocks, red was the relevant color, and in two other blocks green was the relevant color. The order of the relevant colors per two blocks was counterbalanced.

One thousand ms after the onset of the visual cue, a two-pulse (low intensity) or five-pulse (high intensity) intracutaneous electrical stimulus (see [27]) was applied at the participant’s left or right forearm. The number of pulses and stimulation side varied randomly from trial to trial. The relevant or target stimulus intensity (low, corresponding with a two-pulse stimulus, or high, corresponding with a five-pulse stimulus) varied between participants and was kept fixed per participant (see below). Foot pedal presses had to be made when the relevant stimulus intensity occurred on the to-be-attended side, but not when the irrelevant intensity was presented on the to-be-attended side, and also not when stimuli of low or high intensity were presented on the unattended side. No speeded responses were required, instead, participants were emphasized to focus on the accuracy of their responses. The task may be considered as an unspeeded variant of a Go/NoGo paradigm with a low proportion (25%) of Go trials, and a relatively high proportion of NoGo trials (75%), and is in terms of design quite similar to the task with visual stimuli employed by Worden et al. [10]. The white fixation cross turned grey when a response was made. Each trial ended 4,000 ms after onset of the electrical stimulus. Feedback on performance was given on practice trials and after each block of trials.

### Design and procedure

Before the start of the experiment, instructions were presented on the screen. The experiment started after the pretest (see below) in a darkened room, and consisted of four blocks, each containing 96 trials. Short rating sessions (see below) were presented at the start, and after each block. A short block containing 16 practice trials was presented before the start of the third block.

In the sustained spatial attention blocks (block 1+3 or block 2+4, counterbalanced) the relevant color was presented the first half of the block to the left and the second half of the block to the right or vice versa (counterbalanced). In the transient spatial attention blocks (block 2+4 or block 1+3) the side of the relevant color randomly varied over trials. In each block, there were four equally likely events, namely an electrical stimulus of two or five pulses, applied to the left or the right forearm. Each possible event occurred 24 times.

### Pretest

The individual sensation threshold, the pain threshold, and the pain tolerance threshold were obtained in a pretest by increasing the current of a five-pulse stimulus with steps of 0.1 mA starting from zero. Participants were instructed to report the first stimulus that they were able to detect (sensation threshold: 0.3 mA, SE 0.02). With an increasing amplitude, the character of the stimulus changed from a sense of being touched to a more prickling sensation (pain threshold: 0.8 mA ± 0.1). Finally, participants reported when a stimulus became annoying (pain tolerance threshold: 1.2 mA ± 0.2). The amplitudes used during the experiment were set at the individual pain tolerance threshold.

### Rating sessions

The subjective intensity ratings of the employed electrical stimuli were measured at five moments during the experiment. Participant received two low intensity stimuli and two high intensity stimuli to each forearm in a random order in each rating-session. Participants were instructed to rate the stimulus intensity of each stimulus on a 0–10 visual analogue scale (VAS) by using the left and right arrows on a keyboard. The value “0” corresponded to ‘no feeling at all’ whereas “10” corresponded to ‘extremely painful’.

### Apparatus

Two Digitimer DS5 constant current stimulators (Digitimer, Welwyn Garden City, UK) were used to present the electrical stimuli, one for each stimulation side. Bipolar concentric electrodes [27] were placed above the median nerves of the left and right forearms. In the case of sufficiently low intensities (see [28]), these electrodes selectively activate Aδ-fibers located in the epidermis through a tiny needle that is inserted right under the skin.

During the experiment, stimuli of two intensity levels were applied with a fixed current that matched the individual pain tolerance threshold of each participant (see pretest). The low intensity stimuli consisted of a volley of two 1 ms rectangular pulses and the high intensity stimuli consisted of a volley of five 1 ms rectangular pulses. To control for possible temporal summation an interpulse interval between subsequent pulses in the pulse train of 5 ms was chosen, which lies well outside the refractory period [29]. Stimulus presentation, response registration and production of external triggers were controlled by E-prime Software (version 2.0).

### Recordings

EEG was recorded from 61 standard channel positions (extended 10–20 system), using passive Ag/AgCl electrodes mounted on an electrocap (EasyCap GmbH, Herrsching-Breitbrunn, Germany). All electrode impedances were kept below 10 kΩ. A ground electrode was placed on the forehead. The vertical and horizontal electrooculogram (vEOG and hEOG) were measured with bipolar Ag/AgCl electrodes located on the outer canthi of both eyes and from above and below the left eye. Signals passed through a 72 channels QuickAmp amplifier (Brain Products GmbH, Munich, Germany) and were recorded online with an in-built average reference at a sample rate of 500 Hz. Online filtering with a 140 Hz low pass filter and a notch filter of 50 Hz was applied. No online high pass filter was applied.

### Data analysis

#### MBSR training

As individual differences in training time might play a role in observed group differences, we established whether there were possible differences in reported training times after the MBSR training, so before session 1 for the post-post2 group and before session 2 for the pre-post group, and also before session 2 for the post-post2 group. This was assessed with an ANOVA with the between-subjects factor Group.

#### Questionnaire

FFMQ-SF scores obtained at the start of the study, before the MBSR training took place, were first compared between the two groups (pre-post vs. post-post2) with an ANOVA to check whether the random allocation had been successful. Subsequently, the FFMQ-SF scores obtained at the start of the first and second experimental session were evaluated with a repeated measures ANOVA with the within-subjects factor Session and the between-subjects factor MBSR Group. We also examined whether FFMQ-SF scores obtained after the MBSR training were related to the overall duration of the individual training time as reported in the logbooks by including Individual Training Time as a covariate.

#### Behavioral data

Hit and false alarm rates were determined and were subsequently used to compute signal detection theory measures: sensitivity (*d'*) and response bias (*lnβ*) (see [24]). A repeated measures ANOVA was performed on both measures with the within-subjects factors Attention Condition (sustained or transient) and Session (first or second), and the between-subjects factor MBSR Group (pre-post vs. post-post2). Half of the participants had to detect the low and the other half had to detect the high intensity stimulus. To reduce the number of between-subjects factors we ignored this factor in the reported analyses as earlier analyses for the experimental blocks implied no relevant effects including this factor. Reaction times (RT) for correct target detection responses were evaluated with the same factors. We additionally examined whether sensitivity and response bias were related to the Individual Training Time by including this variable as a covariate in the analyses per MBSR Group.

Stimulus intensity ratings assessed with the VAS during the five rating sessions around the experimental blocks were analyzed with a repeated measures ANOVA with Stimulus Intensity (low vs. high), Stimulation Side (left vs. right), Block (0 [before the first experimental block]– 4 [after the final experimental block]), and Session (2) as the within-subject factors, and MBSR Group as the between-subjects factor. We also examined whether there were any changes in pain tolerance thresholds assessed during the pretests with Hand (left or right), and Session as the within-subject factors, and with MBSR Group as the between-subjects factor. Additionally, we examined whether the reported VAS data and pain tolerance thresholds were related to the Individual Training Time by including this variable as a covariate in analyses per MBSR Group.

#### EEG data

EEG was analyzed using Brain Vision Analyzer (Version 2.1.1.327; Brain Products GmbH, Munich, Germany). EEG data were filtered offline (24 dB/oct; high pass: 0.15 Hz; low pass: 20 Hz). Next, relevant segments were extracted from the continuously measured EEG, from 2,200 ms before until 1,500 ms after the onset of the electrical stimuli (which equals the interval from 1,200 ms before until 2,500 ms after the cue). A baseline was set from -1,130 till 1,030 ms before the electrical stimuli, shortly before presenting the cue. Next, trials were removed in which eye movements (> 100 μV) were made in the direction of the hands for the time interval from -1,000 until 200 ms after onset of the electrical stimuli. Trials with major artefacts were removed (gradient criterion: 100 μV/ms; min-max criterion: -/+ 250 μV; low activity criterion: 0.1 μV per 50 ms). Subsequently, Independent Component Analysis (ICA ocular correction) was applied to exclude components that have no cortical origin (e.g., EOG, electromyographic, and electrocardiographic artefacts). Next, two separate analyses were performed, one to examine the orienting phase (from -1,000 to 2,000 ms relative to cue onset: LPS), and one to examine processing of the electrical stimuli (from -100 to 1,500 ms relative to the onset of the stimuli: ERPs).

For the orienting phase, trials were checked for remaining small artefacts (min-max criterion: -/+ 150 μV), which left on average 98.8% of the trials. A baseline was set from -100 to 0 ms relative to cue onset. For the LPS analysis, the procedure was followed as described in the introduction (Eq 1; see also [11]). We extracted the power of two different frequency bands by performing a wavelet analysis (complex Morlet with Gabor normalization, *c* = 5) on the raw EEG. We extracted the lower α_1_ band (Central frequency: 8.9 Hz; Gaussian low—Gaussian high:7.2–10.7 Hz) and the higher α_2_ band (11.7 Hz; 9.4–14.0 Hz). Individual averages for left and right relevant sides for the transient and sustained attention conditions were computed, separately per session. Next, normalized lateralization indices ([ipsilateral-contralateral]/[ipsilateral+contralateral]) were computed for the two frequency bands for the relevant conditions. An average was computed across both relevant sides per frequency band, which results in the LPS.

The following eight electrode pairs were selected for statistical analyses as they overlay the most relevant brain areas related to orienting towards somatosensory stimuli (based on [9]): C4/3 [central], C6/5 [lateral central], CP4/3 [centroparietal], CP6/5 [lateral centroparietal], P4/3 [parietal], P6/5 [occipitotemporal], PO4/3 [occipitoparietal], PO8/7 [occipital]. Statistical analyses were performed for each of six time windows (width 100 ms; from 400 to 1,000 ms).

Each ANOVA implied 64 separate tests per time window, as ANOVAs were carried out with the within-subjects factors Session (first or second), Anterior-Posterior axis (C-CP-P-PO), Lateral-Medial Axis (2), Attention Condition (sustained or transient), Band (α_1_, α_2_), and the between-subjects factor MBSR Group (pre-post vs. post-post2). This implies that a correction of the critical *p*-value had to be adopted. To correct for a Type I error, we used an adjusted critical *p*-value for two successive time windows of 0.01 (*p*_crit_ = √ (0.05/(5 [time windows-1] × 64 [tests per window] = 0.0125)); see [30]). This corrected critical *p*-value implies that effects are only considered significant when the critical *p*-value is crossed for two successive time windows (for comparable procedures, see [9, 23, 30, 31]). We additionally examined the influence of Individual Training Time by including this variable as a covariate in analyses per MBSR Group and Session. Application of the aforementioned procedure (6 time windows and 48 tests per time window) indicated that for these analyses a critical *p*-value of 0.01 for two successive time windows should be applied.

For the analyses performed to evaluate the processing of the intracutaneous electrical stimuli, we also checked for remaining small artefacts, which left on average 98.7% of the trials. A new baseline from -100 to 0 ms relative to the onset of the electrical stimuli was set. Subsequently, ERPs were computed for all relevant conditions and sessions (cued/uncued, two/five pulse stimuli, left/right side, transient/sustained attention condition, and first/second session) for each group. Appropriate time windows and electrodes were selected based on our earlier study in combination with an inspection of the grand average waveforms. We expected to observe the same components as reported in Van der Lubbe et al. (2017 [9]), the N130, N180, and the P340, and comparable effects of attention. Importantly, we expected these effects to be more pronounced after taking part in the MBSR training sessions. We selected the same time windows and the same electrodes, but averaged across left and right stimuli to reduce the number of factors. The N130 was estimated as the mean amplitude within the 120–140 ms interval over the T7 and T8 electrodes. The N180 was measured as the mean amplitude within the 170–190 ms interval over the C5 and C6 electrodes. The P340 was estimated as the mean amplitude from 320 to 360 ms at the Cz electrode. Greenhouse-Geisser *ε* correction of the degrees of freedom was applied whenever appropriate. Again, effects might depend on Individual Training Time. Therefore, this variable was included as a covariate in analyses per MBSR Group and session(s).

## Results

### MBSR training

Analyses of the individual logbooks indicated that during the training period Individual Training Time based on the homework assignments amounted to 1,108 minutes on average (range: 440–1,955 min, SE: 72.). No differences in training times were observed between the pre-post and the post-post2 groups in the session directly after the MBSR training (*F*(1,26) = 1.8, p = 0.186, *η*_*p*_^*2*^ = 0.07; pre-post second session: 1,017 min, SE: 98; post-post2 first session: 1,211 min, SE: 105), although in the second session Individual Training Time for the post-post2 group became larger as compared to the pre-post group (*F*(1,26) = 5.4, p = 0.028, *η*_*p*_^*2*^ = 0.17; post-post2 second session: 1,498, SE: 151). This seems not so surprising as participants were encouraged to continue with the home assignments.

### Questionnaire

Analyses of the FFMQ-SF scores at the start of the study revealed no observable differences between the pre-post (mean: 75.8; 95% confidence interval: [70.6–81.4] and the post-post2 group (79.4 [73.8–84.9]), *F*(1,26) = 0.9, *p* = 0.34, *η*_*p*_^*2*^ = 0.035, which indicates that the random allocation to the groups was successful.

**Fig 3:**
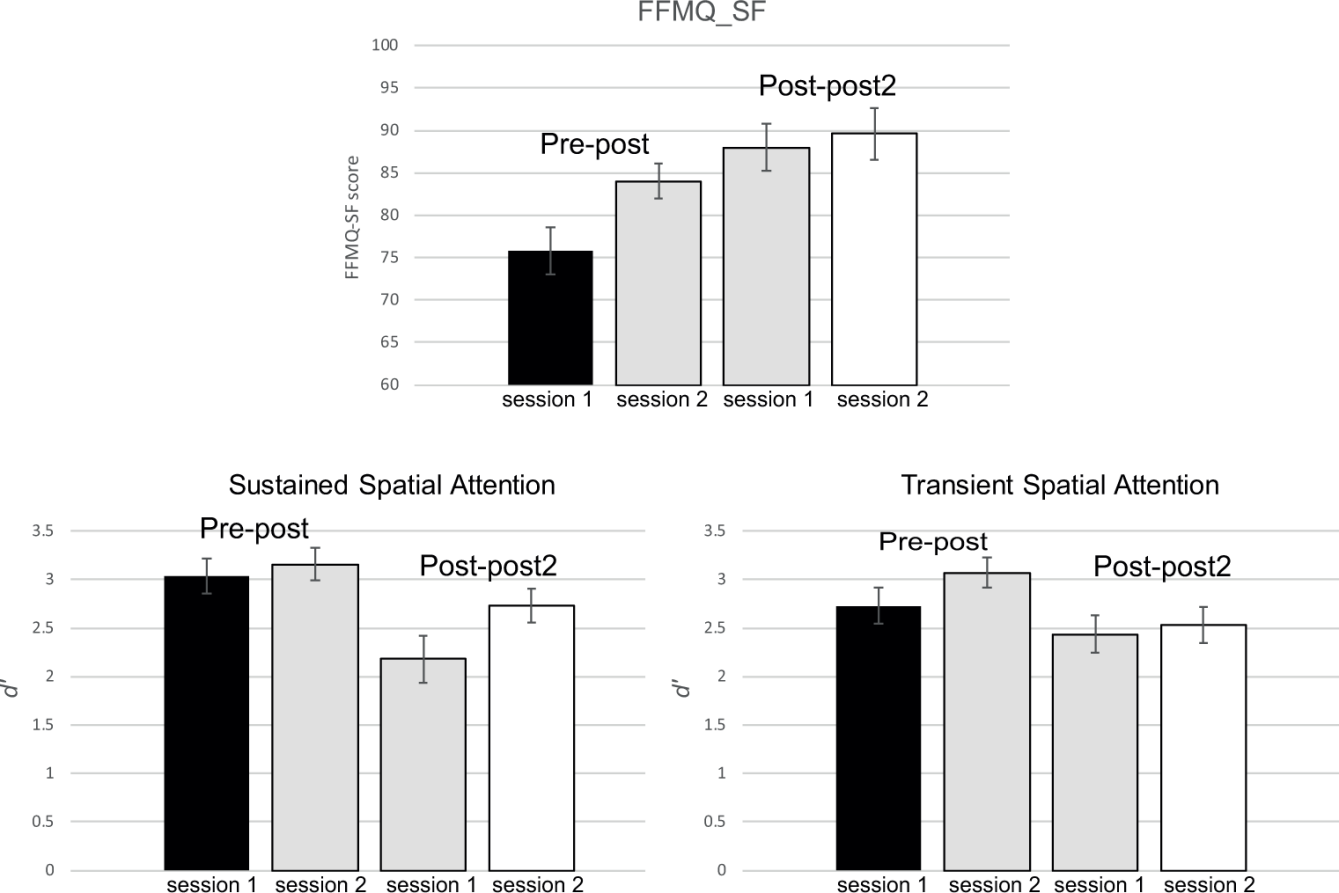
In the upper panel, the obtained average FFMQ-SF (five facet mindfulness questionnaire–short form, see [23]) scores are displayed for both the pre-post and the post-post group at the start of the first and second session. In the lower panels, average sensitivity (*d'*, a measure derived from signal detection theory, see [25]) in detecting the target stimulus intensity of the intracutaneous electrical stimuli during the first and the second session is displayed for both the pre-post and the post-post2 groups for the blocks in which attention had to be consistently directed at one side (sustained spatial attention) and blocks in which the to-be-attended side varied from trial to trial (transient spatial attention). The displayed vertical bars are standard error bars.

The FFMQ-SF scores obtained at the start of the EEG measurement sessions (displayed in Fig 3) showed an effect of Session, *F*(1,26) = 10.9, *p* = 0.003, *η*_*p*_^*2*^ = 0.29, an effect of MBSR Group, *F*(1,26) = 6.4, *p* = 0.018, *η*_*p*_^*2*^ = 0.20, and an interaction between MBSR Group and Session, *F*(1,26) = 4.9, *p* = 0.036, *η*_*p*_^*2*^ = 0.16. Separate analyses per session revealed an effect of MBSR Group for the first session, *F*(1,26) = 9.3, *p* = 0.005, *η*_*p*_^*2*^ = 0.26, with lower scores for the pre-post (75.8 [70.6–81.5]) than for the post-post2 group (88.0 [82.0–94.0]). These findings indicate that in the latter group the MBSR training increased their self-perceived mindfulness. Analyses of the scores obtained in the second session no longer showed differences as a function of MBSR group, *F*(1,26) = 2.4, *p* = 0.134, *η*_*p*_^*2*^ = 0.08. FFMQ-SF scores now suggested increased mindfulness for both groups (pre-post: 84.0 [80.0–87.9]; post-post2: 89.6 [84.2–95.1]). These findings suggest that the effect of the MBSR training on self-report is stable for a duration of eight weeks. Finally, we examined whether the FFMQ-SF scores obtained after the MBSR training for members of both groups were related to the Individual Training Time. A significant positive correlation (Pearson) was found between these variables (*r* = 0.48, *p* = 0.023), indicating that increased training time was reflected in increased self-perceived mindfulness.

### Behavioral data

Participants were well able to discriminate between the relevant and the irrelevant stimulus intensities as the observed mean *d'* scores (see Fig 3) were all clearly much larger than zero. Analyses were performed with Session and Attention Condition (sustained vs. transient) as the within-subjects factors, and with MBSR Group (pre-post vs. post-post2) as the between-subject factors. A main effect of Session was observed, *F*(1,26) = 9.0, *p* = 0.006, *η*_*p*_^*2*^ = 0.26, with an increase in *d'* scores from the first (2.60 [2.35–2.84]) to the second session (2.87 [2.68–3.07]). No difference in sensitivity between the sustained (2.78 [2.54–3.02]) and the transient (2.69 [2.45–2.94]) spatial attention conditions was observed, *F*(1,26) = 0.42, *p* = 0.42, *η*_*p*_^*2*^ = 0.028. A main effect of MBSR Group was observed, *F*(1,26) = 7.4, *p* = 0.012, *η*_*p*_^*2*^ = 0.22, with larger *d'* scores for the pre-post group (3.00 [2.73–3.27]) than for the post-post2 group (2.47 [2.18–2.67]). A significant interaction was observed between Session, Attention Condition, and MBSR Group, *F*(1,26) = 5.4, *p* = 0.031, *η*_*p*_^*2*^ = 0.17. To clarify this interaction, separate analyses were conducted per Attention Condition.

Analyses of the sustained spatial attention condition revealed a main effect of Session, *F*(1,26) = 5.9, *p* = 0.023, *η*_*p*_^*2*^ = 0.18, which again implied an increase in *d'* scores from the first (2.61 [2.30–2.92]) to the second session (2.95 [2.70–3.20]). We also observed a main effect of MBSR Group, *F*(1,26) = 7.5, *p* = 0.011, *η*_*p*_^*2*^ = 0.23, with larger *d'* scores for the pre-post (3.10 [2.77–3.42]) than for the post-post2 group (2.46 [2.11–2.81]), but noticed no interaction between Session and MBSR Group, *F*(1,26) = 2.5, *p* = 0.128, *η*_*p*_^*2*^ = 0.09.

Analyses of the transient spatial attention condition revealed a main effect of Session, *F*(1,26) = 5.2, *p* = 0.030, *η*_*p*_^*2*^ = 0.17, which reflected an increase in *d'* scores from the first (2.58 [2.31–2.86]) to the second session (2.80 [2.55–3.05]). No main effect of MBSR Group, *F*(1,26) = 3.1, *p* = 0.091, *η*_*p*_^*2*^ = 0.11, and no interaction between MBSR Group and Session was observed, *F*(1,26) = 1.7, *p* = 0.202, *η*_*p*_^*2*^ = 0.06.

Analyses of *lnβ* (not displayed) showed no effects involving the factors Session, Attention Condition, and MBSR Group (1.18 [0.67–1.69]). The analyses of the first session for the post-post2 group including the covariate Individual Training Time revealed that the effects obtained on *d'* and *lnβ* measures were not modulated by training time. This conclusion also applied to the results of the second session for the pre-post and the post-post2 group. No significant effects (*p* > 0.10) were observed on RT of the correct target detection responses (1,043 ms [964 – 1,122]), which indicates that participants followed the instruction to take their time to make accurate and unspeeded responses. Additional analyses including the covariate Individual Training Time revealed no significant effects.

**Fig 4:**
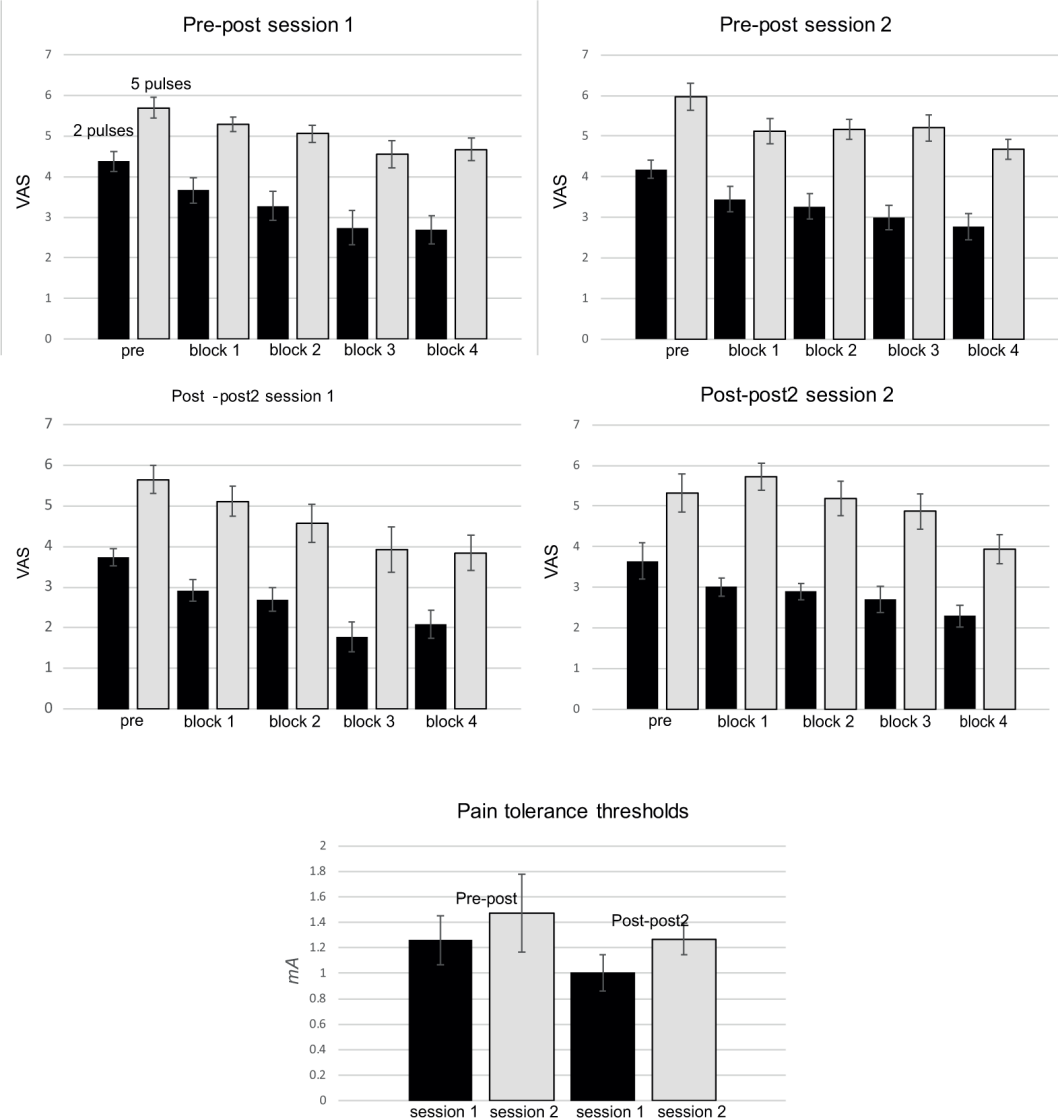
In the upper two panels, the average pain ratings assessed with visual analog scales (VAS) obtained during the short rating sessions are displayed for electrical stimuli with two or five pulses. Scores are indicated per group (pre-post and post-post2) for both sessions. Furthermore, scores were obtained before the start of the session (pre), and after each experimental block (block 1, etc.). In the lower panel, pain tolerance thresholds (in *mA*) are displayed that were obtained during the pretests at the start of the first and the second session for both groups. The displayed vertical bars are standard error bars.

The analyses of the VAS scores (see Fig 4) obtained during the rating sessions revealed a main effect of Block, *F*(4,104) = 31.9, *p* < 0.001, *η*_*p*_^*2*^ = 0.55. Contrast analyses revealed that this effect can be characterized as a linear decrease in reported intensity over time, *F*(1,26) = 56.2, *p* < 0.001, *η*_*p*_^*2*^ = 0.68. Before the first block, participants scored the intracutaneous stimuli as 4.8 [4.5–5.2] while after the fourth block the stimuli were scored as 3.3 [3.0–3.7]. Additionally, an effect of Stimulus Intensity was observed, *F*(1,26) = 237.5, *p* < 0.001, *η*_*p*_^*2*^ = 0.90, which showed that two-pulse stimuli (3.1 [2.7–3.4]) were rated as less painful than five-pulse stimuli (5.0 [4.6–5.3]). Analyses including the covariate Individual Training Time revealed no effect of this variable for the post-post2 group in session 1. For session 2, this analysis revealed an interaction between Individual Training Time and Block, *F*(4,100) = 3.8, *p* = 0.025, *η*_*p*_^*2*^ = 0.13. This effect was also significant when examining the VAS scores before the first block and after the fourth block, *F*(1,25) = 7.6, *p* = 0.011, *η*_*p*_^*2*^ = 0.23. Separate analyses per intensity showed a positive correlation between Individual Training Time and the change across blocks for low intensity stimuli (*r* = 0.45, *p* = 0.016) and a trend effect for high intensity stimuli (*r* = 0.35, *p* = 0.064), suggesting that the reduction in VAS scores over time was smaller when participants had more training time.

Analyses of the pain tolerance thresholds obtained during the pretests (see also Fig 4) revealed a just significant effect of Session, *F*(1,26) = 4.6, *p* = 0.042, *η*_*p*_^*2*^ = 0.15, which reflected higher thresholds in the second session (1.4 mA [1.0–1.7]) than in the first session (1.1 mA [0.9–1.4]). Analyses including the covariate Individual Training Time revealed no additional effects.

### EEG data

To explore whether there were differences in the allocation of attention due to the MBSR training in the sustained and transient attention conditions, we first examined the orienting phase. This was done with the LPS for the lower and the higher alpha bands. The subsequent influence of sustained and transient spatial attention on the processing of the intracutaneous electrical stimuli was examined with ERPs.

#### The orienting phase examined with the LPS

Overall analyses were performed on lateralized power in the α_1_ and α_2_ bands in 100 ms time windows from 400 to 1,000 ms after cue onset with the within-subjects factors Session (first or second), Anterior-Posterior axis (C-CP-P-PO), Lateral-Medial axis (2), Attention Condition (sustained or transient), Band (α_1_, α_2_), and the between-subjects factor MBSR Group (pre-post vs. post-post2). These analyses consistently showed main effects and interactions involving the factors Anterior-Posterior axis, Lateral-Medial axis, Attention Condition, and Band, and most importantly, all analyses revealed interactions between Anterior-Posterior axis, Lateral-Medial axis, Attention Condition, and Band, *F*(3,78) > 11.2, *p* < 0.001, *η*_*p*_^*2*^ > 0.30. These results justify separate explorations per band (see below). Importantly, no systematic effect of MBSR and/or Session was observed. As observed effects might be modulated by Individual Training Time, separate analyses were additionally performed per MBSR group and relevant session(s). These analyses, however, revealed no significant effects or interaction with Individual Training Time. Therefore, this variable was not further examined in the reported analyses per band.

**Fig 5:**
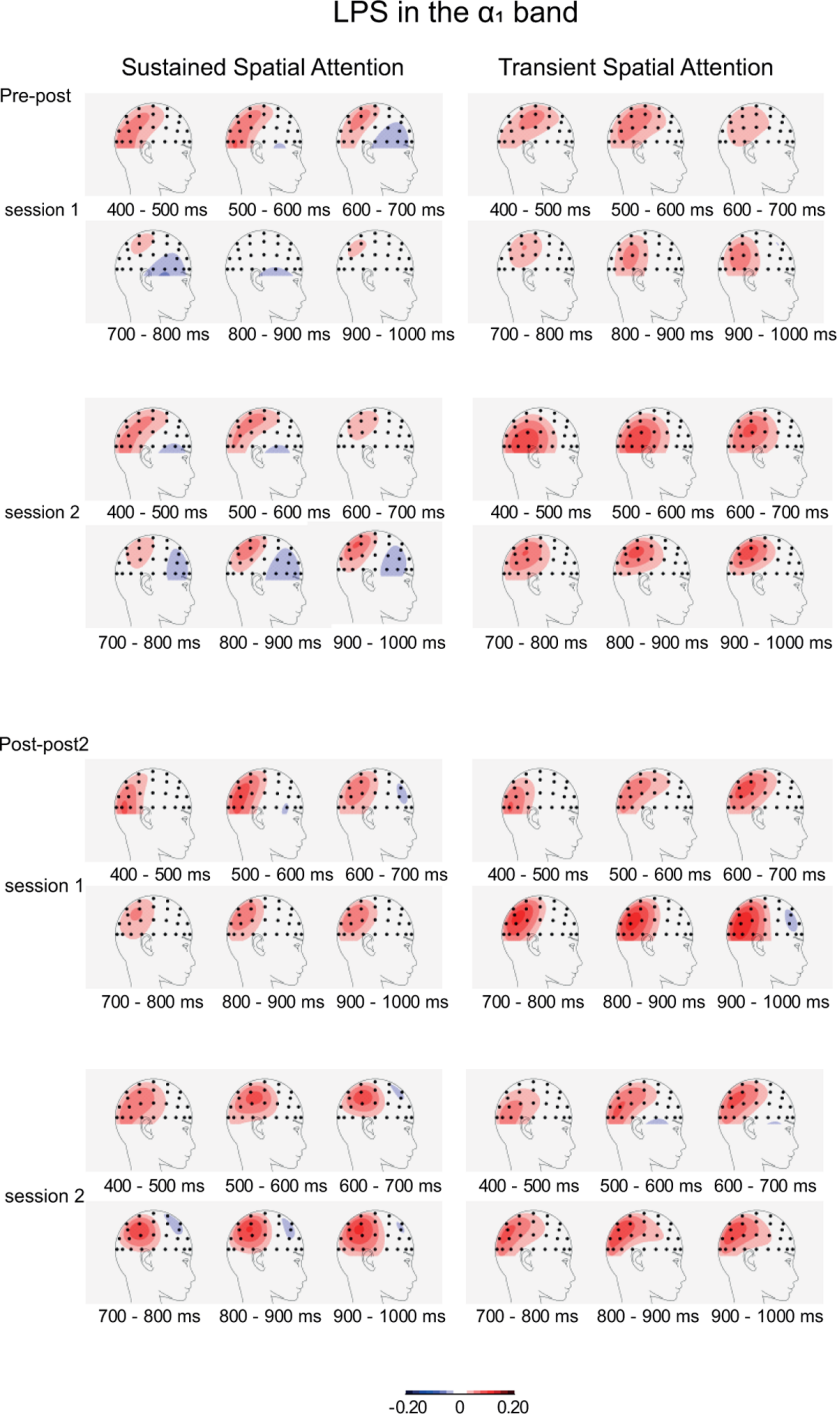
Topographies of lateralized power spectra (LPS) in the lower alpha band (α_1_: 7.2–10.7 Hz) during the orienting phase. The ipsi-contralateral difference of normalized alpha power is projected on the right hemisphere. Topographies were determined for 100 ms intervals from 400 ms after cue onset until onset of the intracutaneous electrical stimulus. The topographies were based on interpolation by spherical splines (4^th^ order). Results are displayed for the pre-post group (the upper panels) and the post-post2 group (the lower panels), for the first and the second session, and for the sustained (left panel) and transient spatial attention conditions (right panel).

**The α**_**1**_ **band.** For this band, we observed interactions between Anterior-Posterior axis, and/or Lateral-Medial axis, and Attention Condition (*p* < 0.01) from 400 to 1,000 ms. Furthermore, consistent interactions were observed between Anterior-Posterior and Lateral-Medial axis (*p* < 0.01). Analyses per electrode pair revealed major effects of Attention Condition, being initially most significant over lateral central sites (C6/5; 400–700 ms) then over parietal and centroparietal sites (P4/3-CP4/3; 700–900 ms) and finally over central sites (C4/3; 900–1,000 ms, *Fs*(1,26) > 23.8, *p* < 0.001, *η*_*p*_^*2*^ > 0.47; see S1 Table). Inspection of these sites (see Fig 5) reveals more positive values in the transient than in the sustained spatial attention condition, so a larger normalized ipsi-contralateral difference in the case of transient spatial attention. Inspection of the intercepts showed initially an effect (i.e., more positive values) over central sites (C4/3; 400–500 ms), then over lateral parietal and lateral centroparietal sites (P6/5-CP6/5; 500–800 ms), and finally over lateral parietal sites (P6/5; 800–1,000 ms, *Fs*(1,26) > 10.1, *p* < 0.005, *η*_*p*_^*2*^ > 0.28; see S2 Table). This pattern is also visible in Fig 5.

**Fig 6:**
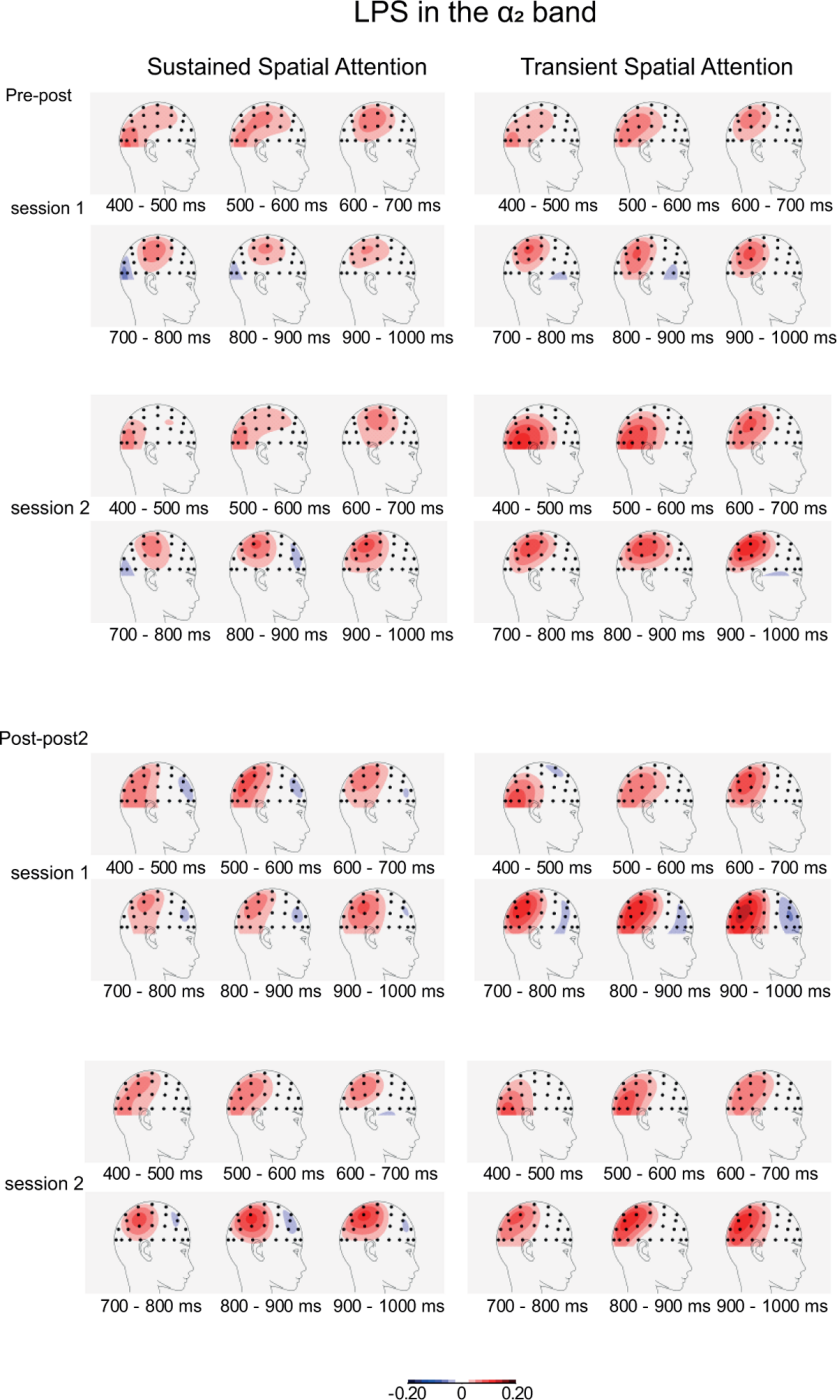
Topographies of lateralized power spectra (LPS) in the higher alpha band (α_2_: 9.4–14.0 Hz) during the orienting phase. For further details, see Fig 5.

**The α**_**2**_ **band.** Again, interactions were observed between Anterior-Posterior axis, and/or Lateral-Medial axis, and Attention Condition (*p* < 0.01) from 400 to 1,000 ms, and additionally, consistent interactions were observed between Anterior-Posterior and Lateral-Medial axis (*p* < 0.01). Separate analyses per electrode pair showed clear effects of Attention Condition. Initially, effects were most significant over lateral occipital sites (PO8/7; 400–500 ms), then over lateral centroparietal and lateral parietal sites (CP6/5-P6/5; 500–800 ms), over parietal sites (P4/3; 800–900 ms), and again over lateral centroparietal sites (CP6/5; 900–1,000 ms; *Fs*(1,26) > 55.0, *p* < 0.001, *η*_*p*_^*2*^ > 0.67; see S3 Table). Inspection of these sites in Fig 6 again suggests increased positive values (i.e., a larger normalized ipsi-contralateral difference) in the transient as compared to the sustained spatial attention condition. Examination of the intercepts revealed initially most significant effects over centroparietal sites (CP4/3; 500–700 ms), then over lateral centroparietal sites (CP6/5; 700–800 ms) and finally over lateral occipital sites (PO8/7; 800–1000 ms; *Fs*(1,26) > 15.6, *p* < 0.001, *η*_*p*_^*2*^ > 0.37; see S4 Table).

#### ERPs related to the intracutaneous electrical stimuli

**Fig 7:**
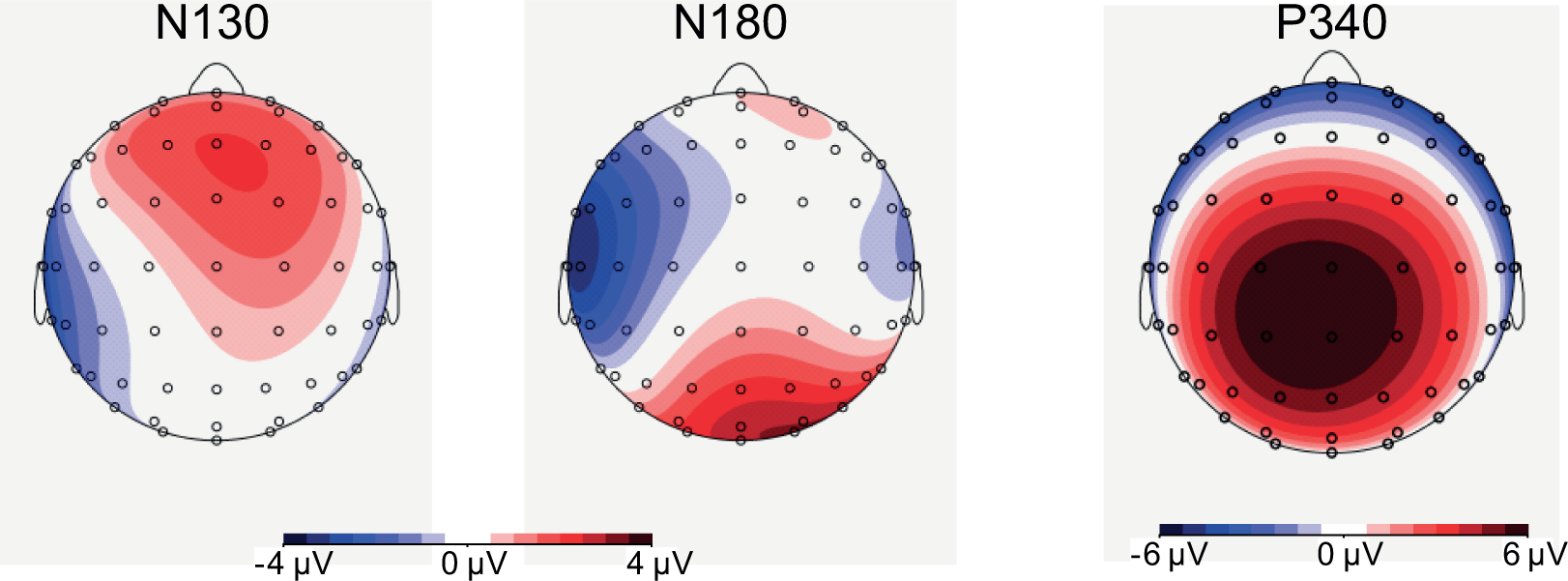
Topographies of the N130, the N180, and the P340 components for the post-post2 group in the first measurement session in the sustained attention condition after presenting the five pulse intracutaneous electrical stimuli at the to-be-attended side. Topographies of the components were quite comparable for both groups in the different conditions, therefore we decided not to display all of them. Displayed results are averaged across left and right electrical stimuli. The left hemisphere displays contralateral activity, while the right hemisphere shows ipsilateral activity. The topographies were based on interpolation by spherical splines (4^th^ order).

The number of factors was reduced by collapsing the data across the left and right electrical stimuli. Inspection of the topographical maps (see Fig 7) confirmed that a lateralized central negativity (N130) with a contralateral maximum over T7/T8 was followed by a lateralized central negativity (N180) that had its maximum over C5/C6. Subsequently, a central P340 was maximal over Cz.

#### The N130 over T7/T8

**Fig 8:**
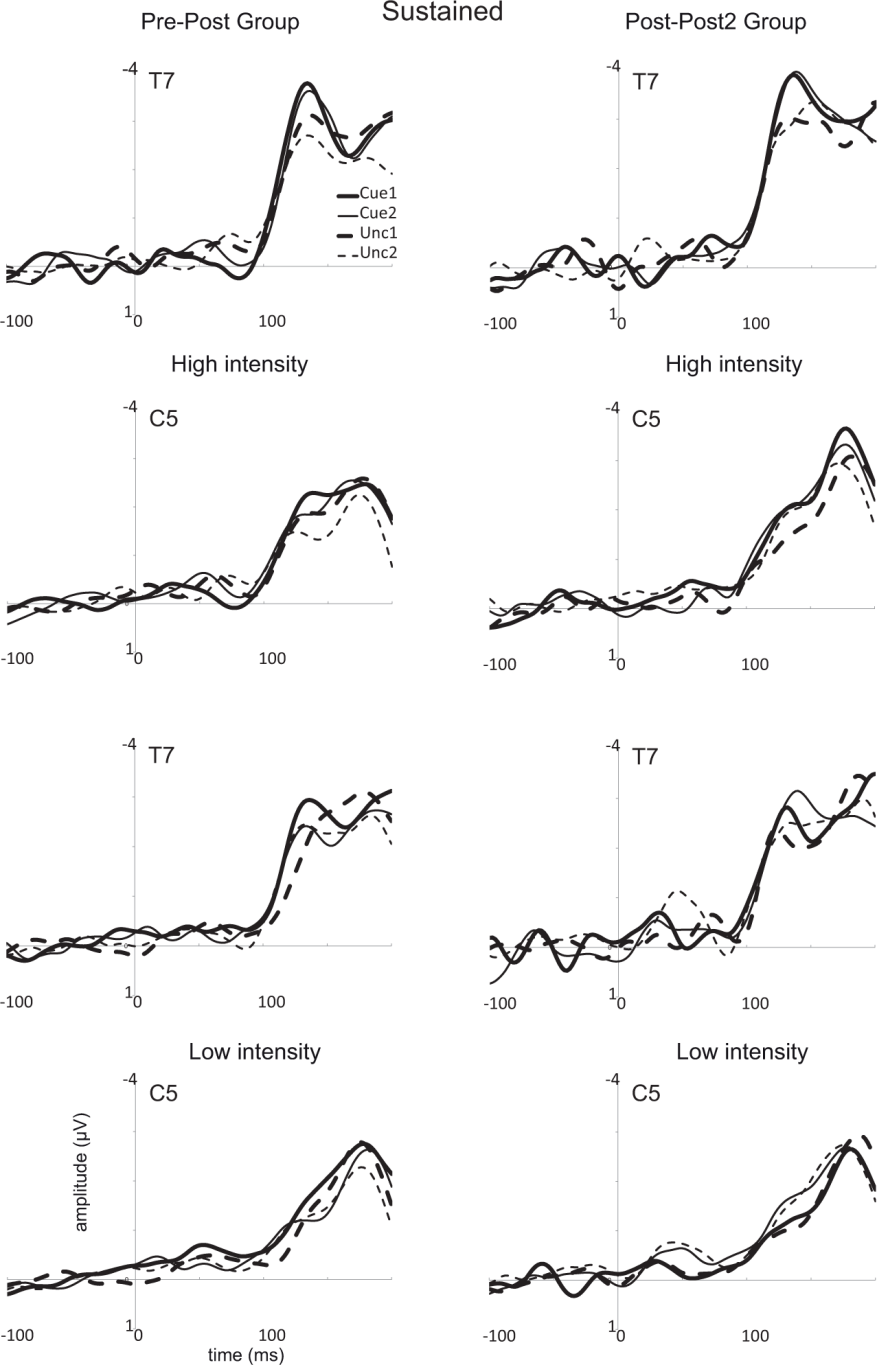
Event-related potentials (ERPs) evoked by the intracutaneous electrical stimuli over contralateral sites (T7 and C5) in the sustained spatial attention conditions. Averages are shown for cued stimuli in the first and the second session (Cue1 and Cue2) and for uncued stimuli in both sessions (Unc1 and Unc2). Results for high intensity stimuli are displayed in the two upper rows while results for the low intensity stimuli are presented in the two lower rows. Results for the pre-post group are presented in the left panel, while results for the post-post2 group are presented in the right panel.

**Fig 9:**
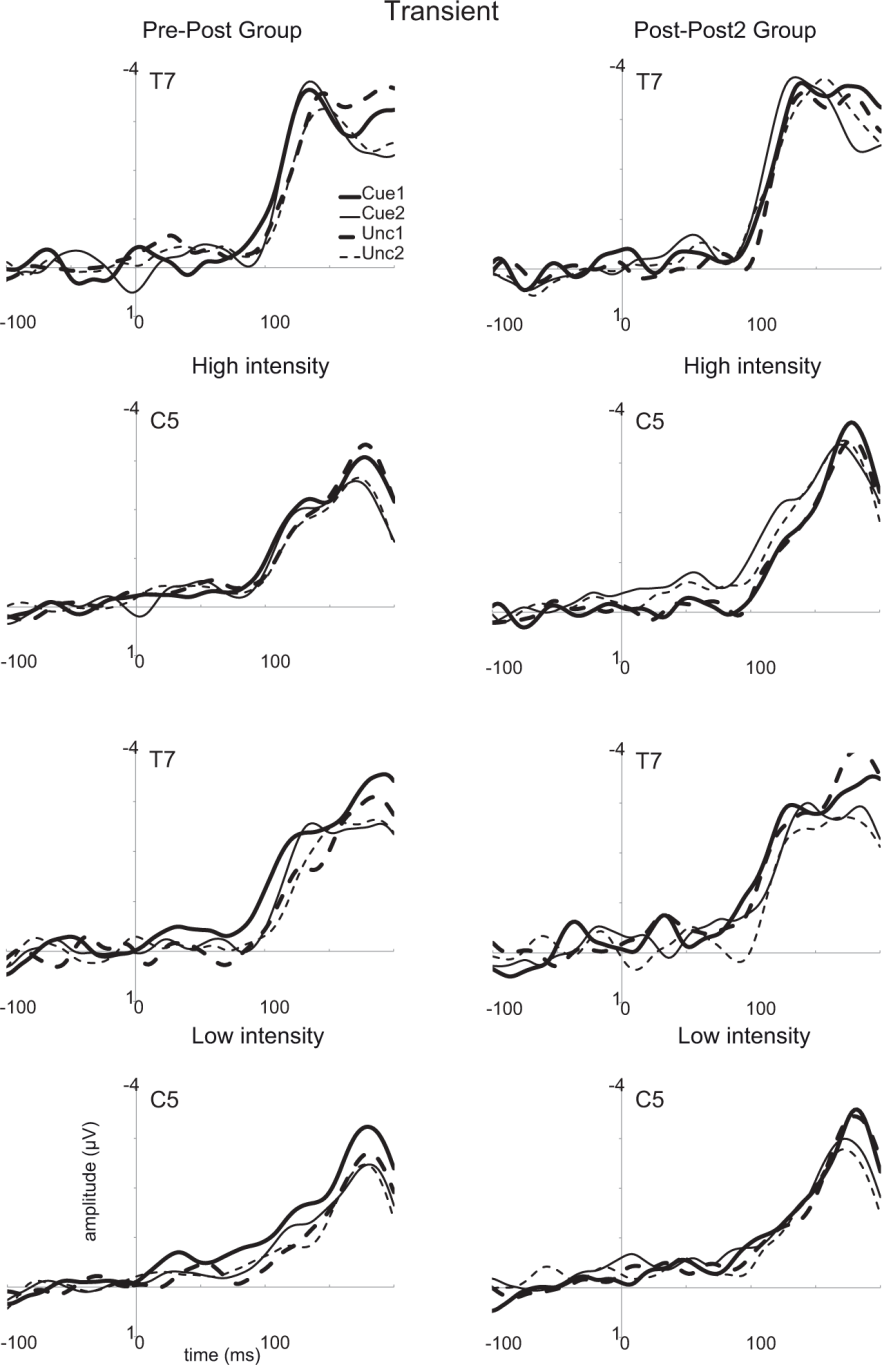
Event-related potentials (ERPs) evoked by the intracutaneous electrical stimuli over contralateral sites (T7 and C5) in the transient spatial attention conditions. For further details, see Fig 8.

A main effect of Electrode was observed, *F*(1,26) = 104.3, *p* < 0.001, *η*_*p*_^2^ = 0.80, revealing more negative amplitudes on contralateral (see Figs 8 and 9) than on ipsilateral (not displayed) electrodes (-2.7 [-3.3 –-2.2] vs. -1.1 μV [-1.4–0.7]). A main effect of Stimulus Intensity was observed, *F*(1,26) = 27.0, *p* < 0.001, *η*_*p*_^2^ = 0.51, and this effect also interacted with Electrode, *F*(1,26) = 21.9, *p* < 0.001, *η*_*p*_^2^ = 0.46. The effect of Stimulus Intensity was present on contralateral (low: -2.3 [-2.8 –-1.8]; high -3.1 μV [-3.7 –-2.6]) but not on ipsilateral electrodes (low: -1.0 [-1.3 –-0.6] vs. high: -1.2 μV [-1.6 –-0.8]). No effect of Attention Condition was observed, *F*(1,26) < 0.1.

A main effect of Cue was observed, *F*(1,26) = 57.2, *p* < 0.001, *η*_*p*_^2^ = 0.69, with more negative amplitudes for cued than for uncued stimuli (-2.1 [-2.6 –-1.7] vs. -1.7 μV [-2.0 –-1.3]). No main effects or significant interactions were found for MBSR Group (*p* > 0.16). Analyses including the covariate Individual Training Time revealed a just significant interaction between this variable and Stimulus Intensity for the post-post2 group in session 1, *F*(1,11) = 5.4, *p* = 0.04, *η*_*p*_^2^ = 0.33. This interaction reflected a decreasing difference between high and low intensity stimuli in the case of more training time (*r* = 0.574, *p* = 0.04). However, no such effect was observed for session 2, *F*(1,11) = 0.3. Furthermore, no effect of Individual Training Time was found for the pre-post group in session 2, *F*(1,13) = 0.3.

#### The N180 over C5/C6

Analyses were performed with the same factors as for the N130, but now C5 (contralateral) and C6 (ipsilateral) were used as levels for the factor Electrode. A main effect of Electrode was observed, *F*(1,26) = 51.9, *p* < 0.001, *η*_*p*_^2^ = 0.67, revealing more negative amplitudes over contralateral (see Figs 7 and 8) than over ipsilateral (not displayed) electrodes (-2.8 [-3.5 –-2.2] vs. -0.9 μV [-1.2 –-0.7]).

A main effect of Stimulus Intensity, *F*(1,26) = 10.5, *p* < 0.005, *η*_*p*_^2^ = 0.29, (low: -1.8 [-2.1 –-1.4]; high -2.0 μV [-2.4 –-1.5]), and a main effect of Cue, *F*(1,26) = 11.0, *p* < 0.005, *η*_*p*_^2^ = 0.30, (cued: -2.0 [-2.4 –-1.6]; uncued -1.7 μV [-2.1 –-1.3]) was observed. We also found a main effect of Session, *F*(1,26) = 8.0, *p* < 0.01, *η*_*p*_^2^ = 0.24, which reflected an overall decrease in amplitudes in the second session (session 1: -2.0 [-2.5 –-1.5]; session 2: -1.7 μV [-2.1 –-1.4]). An interaction was found between Session and Attention Condition, *F*(1,26) = 4.9, *p* < 0.04, *η*_*p*_^2^ = 0.16. A trend to an interaction between Session, Stimulus Intensity, and MBSR Group, *F*(1,26) = 3.5, *p* = 0.072, *η*_*p*_^2^ = 0.12, and a significant interaction between Electrode, Stimulus Intensity, and MBSR Group was observed, *F*(1,26) = 6.7, *p* = 0.016, *η*_*p*_^2^ = 0.21. Additional trends to an interaction with the factor MBSR Group were observed involving the factors Stimulus Intensity, Cue, Attention Condition, and Session, *F*(1,26) > 3.1, *p* < 0.09, *η*_*p*_^2^ > 0.11. Finally, we found an interaction between Session, Stimulus Intensity, Attention Condition, and Cue, *F*(1,26) = 5.6, *p* = 0.025, *η*_*p*_^2^ = 0.18. To clarify these (trend) effects, we first performed a separate analysis of amplitudes over the contralateral electrode, as amplitudes can be expected to be largest on this site. Most importantly, we also performed separate analyses of both sessions as session-specific predictions were presented in our introduction regarding the effect of MBSR Group.

The analysis of amplitudes over the contralateral electrode also revealed an effect of Session, *F*(1,26) = 5.2, *p* = 0.031, *η*_*p*_^2^ = 0.17, and an interaction between Stimulus Intensity, Cue, and MBSR Group, *F*(1,26) = 5.2, *p* = 0.031, *η*_*p*_^2^ = 0.17. It appeared that the pre-post group showed a cuing effect for low intensity stimuli (cued: -2.7 μV; uncued: -2.5 μV) but not for high intensity stimuli (cued: -2.6 μV; uncued: -2.6 μV), while the post-post2 group showed a cuing effect for high intensity (cued: -3.4 μV; uncued: -3.1 μV) but not for low intensity stimuli (cued: -2.9 μV; uncued: -2.8 μV).

The analysis of the first session showed main effects of Electrode, Stimulus Intensity, Cue, and Attention Condition, *F*(1,26) > 5.4, *p* < 0.027, *η*_*p*_^2^ = 0.17. Furthermore, interactions were observed involving the factor MBSR Group (Electrode by Stimulus Intensity by MBSR Group; Stimulus Intensity by Attention Condition by MBSR Group; Electrode by Stimulus Intensity by Cue by MBSR Group; *F*(1,26) > 5.2, *p* < 0.031, *η*_*p*_^2^ = 0.17). Separate analyses per MBSR Group did not reveal significant effects involving the factors Cue and Attention Conditions, providing no clear explanation for the earlier observed (trend) effects. We also observed an interaction between Stimulus Intensity, Attention Condition, and Cue, *F*(1,26) = 6.3, *p* = 0.019, *η*_*p*_^2^ = 0.19. The effect of Cue appeared larger for low intensity stimuli in the transient than in the sustained attention condition (-0.4 vs. -0.1 μV), while an opposite pattern seemed present for high intensity stimuli (-0.2 vs. -0.5 μV).

The analysis of the second session revealed main effects of Electrode, Stimulus Intensity, and Cue, *F*(1,26) > 7.3, *p* < 0.012, *η*_*p*_^2^ = 0.22. No interactions were observed involving the factor MBSR Group (*p* > 0.10). A just significant interaction was observed between Electrode, Stimulus Intensity, Attention Condition, and Cue, *F*(1,26) = 4.3, *p* = 0.048, *η*_*p*_^2^ = 0.14. Analyses of the amplitudes on the contralateral electrode revealed no significant effects (*p* > 0.14), while analyses of the ipsilateral electrode revealed significant effects of Stimulus Intensity and Cue, and a significant interaction between Stimulus Intensity, Attention Condition, and Cue, *F*(1,26) > 4.3, *p* < 0.05, *η*_*p*_^2^ > 0.14. The effect of Cue was now smaller in the transient than in the sustained spatial attention condition for low intensity stimuli (-0.2 vs. -0.6 μV) while for high intensity stimuli it was larger in the transient than in the sustained spatial attention condition (-0.4 vs. -0.1 μV).

Analyses including the covariate Individual Training Time revealed a just significant main effect of this variable for the post-post2 group in the second session, *F*(1,11) = 5.2, *p* = 0.043, *η*_*p*_^2^ = 0.32. The longer the training time, the smaller was the observed amplitude of the N180 (*r* = -0.567, *p* = 0.043). An interaction was also observed between Individual Training Time, Electrode, and Stimulus Intensity, *F*(1,11) = 5.2, *p* = 0.044, *η*_*p*_^2^ = 0.32, but separate analyses of the contra- and ipsilateral electrodes revealed no effects. No effects involving the covariate Individual Training Time were observed for this group in the first session, and no effect was observed for the pre-post group in the second session.

#### The P340 at Cz

Analyses were performed with Cue (cued, uncued), Stimulus Intensity (low, high), Attention Condition (sustained, transient), and Session as the within-subjects factors, and with MBSR Group (pre-post, post-post2) as the between-subjects factor.

**Fig 10:**
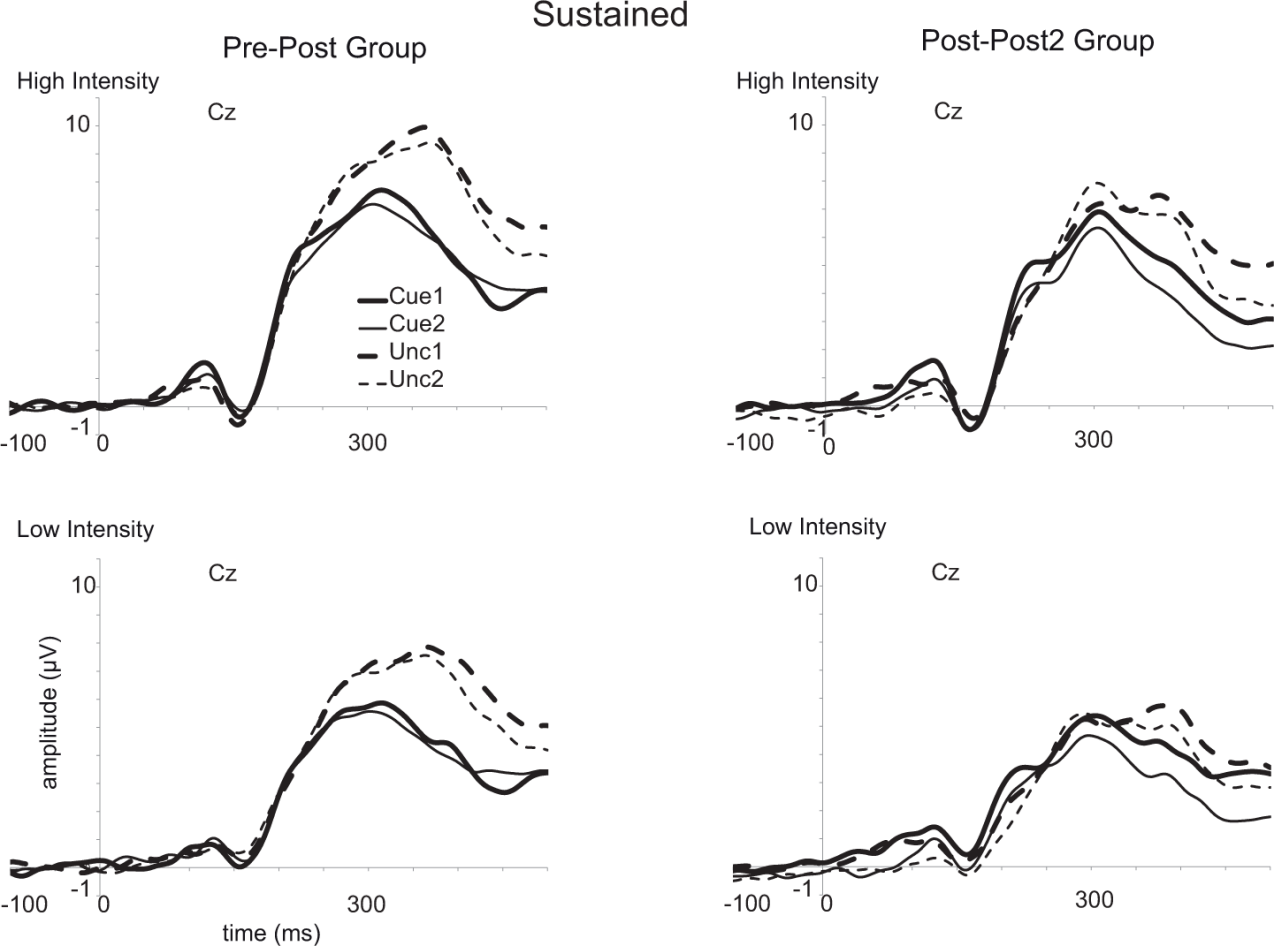
Event-related potentials (ERPs) evoked by the intracutaneous electrical stimuli at the vertex (Cz). Results are shown for the sustained spatial attention condition. Averages are shown for cued stimuli in the first and the second session (Cue1 and Cue2) and for uncued stimuli in both sessions (Unc1 and Unc2). Results for high intensity stimuli are displayed in the upper row while results for the low intensity stimuli are presented in the lower row. Results for the pre-post group are presented in the left panel, while results for the post-post2 group are presented in the right panel.

**Fig 11:**
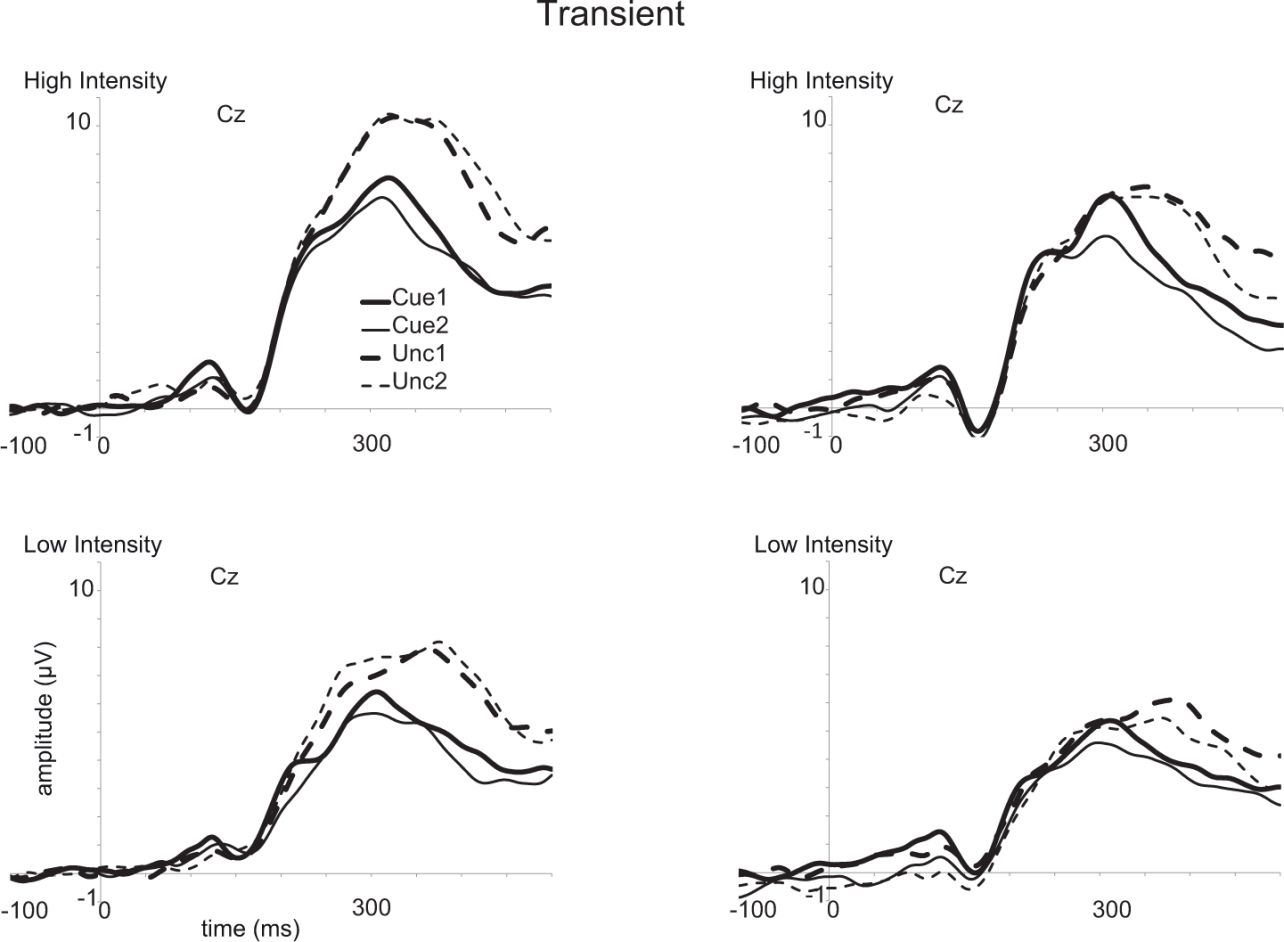
Event-related potentials (ERPs) evoked by the intracutaneous stimuli at the vertex (Cz). Results are shown for the transient spatial attention condition. For further details, see Fig 10.

A main effect of Stimulus Intensity (see Figs 10 and 11) was observed, *F*(1,26) = 79.3, *p* < 0.001, *ηp*^*2*^ = 0.75, revealing larger amplitudes for high intensity (7.5 [6.3–8.7]) than for low intensity (5.6 μV [4.7–6.6]) stimuli. A main effect of Cue was obtained, *F*(1,26) = 34.5, *p* < 0.001, *ηp*^*2*^ = 0.57, which reflected higher amplitudes for uncued (7.5 [6.3–8.6]) than for cued stimuli (5.6 [4.6–6.6]). The effect of Cue interacted with Session, *F*(1,26) = 6.3, *p* < 0.02, *ηp*^2^ = 0.19. The difference in the P340 for cued as compared to uncued stimuli was smaller in the first session (cued: 6.0 μV [4.9–7.1]; uncued: 7.6 μV [6.2–8.9]) than in the second session (cued: 5.3 μV [4.3–6.2]; uncued: 7.4 μV [6.3–8.5]). This interaction seems related to a reduction of the P340 for cued stimuli in the second relative to the first session, *F*(1,26) = 6.1, *p* = 0.02, *ηp*^2^ = 0.19; and not related to a reduction of the P340 for uncued stimuli in the second relative to the first session, *F*(1,26) = 0.2.

An interaction between Cue and Stimulus Intensity, *F*(1,26) = 10.9, *p* = 0.003, *η*_*p*_^2^ = 0.30, showed a larger difference between cued and uncued stimuli for high intensity (cued: 6.4 μV [5.2–7.5]; uncued: 8.6 μV [7.3–9.9]) than for low intensity stimuli (cued: 4.9 μV [4.0–5.8]; uncued: 6.4 μV [5.4–7.4]).

A trend to a significant difference was observed for MBSR group, *F*(1,26) = 3.1, *p* = 0.088, *η*_*p*_^2^ = 0.11, with somewhat larger amplitudes for the pre-post group (7.4 μV [6.0–8.8]) than for the post-post2 group (5.7 μV [4.2–7.2]). Importantly, no interaction was observed between MBSR Group and Session, *F*(1,26) < 0.1. Finally, a trend to an interaction was observed between MBSR Group and Cue, *F*(1,26) = 3.5, *p* = 0.071, *η*_*p*_^2^ = 0.12. The pre-post group showed a larger difference between cued and uncued stimuli (cued: 6.2 μV [4.9–7.6]; uncued: 8.7 μV [7.1–10.2]), while this difference was smaller for the post-post2 group (cued: 5.0 μV [3.6–6.5]; uncued: 6.3 μV [4.6–8.0]).

Analyses including the covariate Individual Training Time revealed a significant relation between this variable and Stimulus Intensity, and also between Stimulus Intensity and Attention Condition for the post-post2 group in the first measurement session, *F*(1,11) > 6.4, *p* < 0.03, *η*_*p*_^2^ > 0.36. Separate analyses per attention condition revealed a significant relation between Individual Training Time and Stimulus Intensity in the transient attention condition, *F*(1,11) > 9.7, *p* = 0.01, *η*_*p*_^2^ = 0.47, but no relation in the sustained attention condition, *F*(1,11) = 1.6. Correlational analyses for the transient attention condition revealed that the difference between high and low intensity stimuli decreased with longer training times (*r* = -0.69; *p* = 0.01).

In the second session, we observed a nearly significant relation between Stimulus Intensity, Attention Condition and Individual Training Time for the post-post2 group, *F*(1,11) = 4.4, *p* = 0.06, *η*_*p*_^2^ = 0.28, but we found no relation between Individual Training Time and Stimulus Intensity when separate tests were performed per attention condition (*p* > 0.35). However, for the sustained attention condition we observed a main effect of Individual Training Time, *F*(1,11) = 5.3, *p* = 0.04, *η*_*p*_^2^ = 0.32. Correlational analyses revealed that amplitudes of the P340 became larger in the case of longer individual training times (*r* = 0.57, *p* = 0.04)

Analyses of the pre-post group in the second measurement session revealed a significant relation between Individual Training Time and Cue, *F*(1,13) = 8.1, *p* = 0.014, *η*_*p*_^2^ = 0.38. The difference in the P340 between uncued and cued stimuli became smaller in the case of larger individual training time (*r* = -0.62, *p* = 0.014).

## Discussion

An intriguing hypothesis regarding the effectiveness of the MBSR training holds that it improves attentional skills by modulating thalamo-cortical loops that selectively affect the sensitivity of relevant sensory cortical areas, like the somatosensory cortex [2]. This modulation within somatosensory areas is thought to involve the inhibitory alpha (α) rhythm of the EEG. This inhibitory mechanism might subsequently affect the processing of intracutaneous electrical stimuli, which may lead to a reduction in pain sensations [6]. In the current study, EEG was measured in an endogenous cuing paradigm to assess changes in attentional orienting, and changes in the processing of the intracutaneous electrical stimuli by computing LPS for the lower and higher α bands, and by computing ERPs elicited by the electrical stimuli. Two versions of the cuing paradigm were employed, which enabled us to examine effects of both sustained and transient spatial attention. Participants were randomly assigned to two groups. In the pre-post group, participants took part in the EEG experiment before (session 1) and directly after the MBSR training (session 2), while in the post-post2 group they participated directly after the training (session 1), and another time eight weeks later (session 2). We predicted that the effect of the MBSR training would be reflected in group differences in the first session, while no differences were expected in the second session due to stable changes caused by the training.

The analyses of individual training times revealed quite large individual differences, and showed that in the second session the total individual training time became larger for the post-post2 group than for the pre-post group. If the individual training time has an additional modulatory effect, then the consequence might be a group difference in mindfulness in the second session albeit reduced as compared to the first session. Scores on the FFMQ-SF test [22] indicated that there were no group differences in self-reported mindfulness before participants took part in the MBSR training. Importantly, a group difference was observed directly before the first EEG measurement session (see Fig 3), while it disappeared at the start of the second EEG measurement session. These findings indicate that the MBSR training had a clear impact on subjective measures of mindfulness, and this effect was stable for approximately eight weeks (for some related recent findings with the FFMQ-SF test, see [32–35]). Although the obtained difference in individual training time between the two groups did not result in group differences in self-reported mindfulness, correlational analyses revealed that larger individual training times resulted in increased mindfulness, suggesting that this variable might have some impact on our other measures. One could argue that the effects on FFMQ-SF scores are due to response bias, as participants that took more time may be more willing to report increased mindfulness. This explanation might also hold for the group difference in the first session and the absence of a group difference in the second session. As some other effects of individual training time were found (see below), we think that this explanation is insufficient.

The analyses of our psychophysical data (*d'* and *lnβ*) revealed that both groups were better in detecting the target stimulus intensity in the second session as *d'* became clearly larger. The pre-post group had in general higher *d'* scores than the post-post2 group, although separate analyses per attention condition only showed a group difference in the sustained and not in the transient spatial attention conditions. The current findings suggest that perceptual sensitivity became larger due to repeated exposure to the intracutaneous electrical stimuli. Effect sizes for both groups were comparable, thus, the predicted selective improvement due to MBSR was not observed. Obviously, larger sensitivity of the pre-post group cannot be ascribed to differences in training time, as this group trained less than the post-post2 group. Together, these behavioral data do not confirm the idea that MBSR training improves attention and thereby the ability to distinguish between different stimulus intensities. Possibly, these effects are masked by the major learning effects due to repeated exposure, the general difficulty of the detection task due to habituation, and/or accidental differences between the pre-post and the post-post2 group. The analyses of the perceived painfulness of the employed stimuli during the rating sessions before and after each block revealed effects of stimulus intensity, and habituation of the perceived intensities. Examination of the influence of individual training time showed an effect for the post-post2 group in the second session, as the decrease in perceived intensity over time was smaller in the case of longer training times. Pain tolerance thresholds obtained during the pretests were slightly higher in the second session, but no effect of the MBSR training was observed in the first session. Thus, apart from the increase in self-reported mindfulness and the influence of individual training time on mindfulness, our behavioral effects did not confirm the hypothesis that participants were better able to focus their attention after the MBSR training.

The analyses of the LPS in the lower alpha band (α_1_) revealed decreased contralateral power over centro-parietal sites, which may be understood as the electrophysiological correlate of directing attention on the relevant forearm in the cue-target interval. This observation extends the earlier findings of studies with visual stimuli (e.g., see [10–12]) to somatosensory stimuli. An interesting observation is that this reduction in power in the transient spatial attention conditions seems to extend to occipital sites (see Fig 5), which may point to the additional involvement of an external (visually based) reference frame while directing attention (for more direct support see [23]), which may be due to the use of visual cues. Major differences between the two conditions suggest that the electrophysiological correlate of orienting was more pronounced in the transient than in the sustained spatial attention condition. This effect may be ascribed to the constant need to reorient in this condition. No support was found for the idea that mindfulness training affects the α_1_ band. Nevertheless, inspection of Fig 5 shows a pattern in the Sustained Attention condition that seems in line with the predictions. Future studies may take this observation into account when formulating their predictions regarding the effect of MBSR training.

The analyses of the higher alpha band (α_2_) also revealed decreased contralateral power especially over centro-parietal sites (Fig 6). Again, a more occipital focus seems present in the transient attention condition and a general difference between the two conditions suggest that orienting was stronger in this condition. No group differences were present for the α_2_ band.

Together, the results of our LPS analyses suggest that orienting in the case of sustained spatial attention is less visually based than in the case of transient shifts. The effect of transient spatial attention above visual areas may be due to the need to use the visual cues, while visual attention becomes less relevant in the sustained attention condition as the relevant side is already known. Furthermore, orienting seems more pronounced in the transient spatial attention condition, possibly due to the need to reorient in this condition. No support was obtained for improved attentional orienting after the MBSR training.

The earliest processing of the intracutaneous electrical stimuli was reflected in the N130 component (see Figs 7–9). The N130 component was clearly more negative for cued than for uncued stimuli and for more intense than for less intense stimuli, which replicates and extends the findings from our previous studies [9,16]. As the N130 likely arises from primary and/or secondary somatosensory areas (e.g., see [16]), it can be concluded that spatial attention affects the processing of intracutaneous electrical stimuli at an early processing level. The cuing effect was not modulated by Attention Condition. Therefore, these findings suggest that there are no differences in the effectiveness between sustained and transient attention conditions, at least at this processing level (see [9]). Although no main difference between the two groups was observed, individual training time had some significant effect. For example, in the first session of the post-post2 group, the difference between low and high intensity stimuli diminished with more training time.

A second component, which had a slightly more medial topography (see Fig 7), peaked around 180 ms (see Fig 8 and 9), and was denoted as the N180. This component had again a clear contralateral focus. The N180 was also larger for cued than for uncued stimuli, larger for more intense than less intense stimuli, and larger in the first than in the second measurement session. In the first session, the N180 was larger in the transient than in the sustained condition, but this effect disappeared in the second session. Thus, our earlier observations regarding some subtle differences in the processing of intracutaneous electrical stimuli between the sustained and transient spatial attention conditions (see [9]) are not stable across different measurement sessions. Possibly, these effects are due to increased familiarity with the testing procedure and/or habituation effects specific for painful stimuli, as the N180 was smaller in the second than in the first measurement session. Although some (trend) effects were observed concerning group and session-related effects none of the effects seemed in line with the predicted effects of the MBSR training. For example, we observed that the pre-post group showed an influence of spatial attention on low intensity stimuli but not on high intensity stimuli, while the post-post2 group showed a reversed effect. In the second session, individual training time had some influence on the amplitude of the N180, as for the post-post2 group the N180 became smaller with longer training time.

The last ERP component that we focused on is the central P340 (see Fig 7, [10–11]), which may be considered as a manifestation of the P3a component. In our earlier studies [9,16,23], we argued that this component reflects a “call for attention” rather than response inhibition because responses are only required on a small proportion of trials and as relevant stimulus intensity had no effect (see [9]). We replicated the effects observed in our earlier studies: the P340 was larger for uncued than for cued stimuli, and larger for more intense than for less intense stimuli. Interestingly, we observed an interaction between Cue and Session; the P340 for cued stimuli was smaller in the second than in the first session, while the P340 for uncued stimuli had about the same amplitude in both sessions. Most relevant for our hypothesis regarding the effect of the MBSR training were some trend effects that seemed only partly in line with the predictions as no session-specific effects were observed regarding group differences. For example, the pre-post group showed larger amplitudes and the difference between cued and uncued stimuli tended to be larger for this group, but these effects were unaffected by session. With regard to individual training time, we observed that the difference between high and low intensity stimuli became smaller with more training for the post-post2 group in the first session, while in the second session a main effect of individual training time for the post-post2 group was observed, which actually revealed that the P340 became larger in the case of longer training. The pre-post group also showed an effect of training time in the second session as the difference between cued and uncued stimuli became smaller with longer training.

None of our observed findings, with the exception of the self-reported mindfulness scores obtained with the FFMQ-SF questionnaire, point to a strong modulatory effect of the MBSR training on observed attentional effects. Nevertheless, the conclusion that MBSR does not modulate attention according to the mechanism proposed by Kerr et al. [2], by selective modulation of thalamo-cortical loops reflected in local changes in the alpha rhythm, seems premature. One aspect that clearly played a role in our study is individual differences in following the homework assignments. Although we thought that major effects of the training would be related to participation in the group sessions, it appeared that individual training time influenced our results and, for example, reduced intensity effects on the P340. One consequence of individual differences might be that the predicted absence of a group effect in the second session was not observed. This might for example explain why the nearly significant difference in the amplitudes of the P340 between the pre-post and the post-post2 group was not modulated by session. Given the influence of individual training time, the choice of our design may be less optimal as the intervention effect is not restricted to the weekly group sessions. For future studies, it may be a good idea to add an earlier measurement session. One group would then receive the intervention between the first and the second session, and a second group between the second and the third session. Furthermore, a third group could be added which would receive a control intervention between the first and the second session, e.g., relaxation by physical exercise. In that case, no group difference would be expected in the first session, while a group difference due to the MBSR training would be expected in the second session. The third session would allow researchers to see whether the group difference disappears, which would demonstrate that observed effects cannot be ascribed to coincidental differences between the groups. Finally, the added control group should reveal that the MBSR effect is selective by showing group differences in the second and the third session.

A recent study by Tsai et al. [21] reported electrophysiological evidence for the view that meditation improves the ability to suppress distracting visual information. This observation may be related to the trend effects that we found for the P340. Thus, with regard to the underlying mechanism responsible for the effect of meditation training, there seems to be some evidence that supports the idea that distraction by to-be-ignored stimuli is reduced, which seems related to attentional control exerted by the anterior cingulate cortex (see [16]). However, no evidence has been observed for the hypothesis that meditation training affects spatial attention, which may be regulated by thalamo-cortical loops to relevant sensory areas, being reflected in the alpha rhythm [2]. Nevertheless, a recent review [36] of Lomas et al. (2015) including a total of 1715 participants suggests that there is in general an increase in alpha power due to mindfulness training, which they associated with a state of relaxed alertness. This observation gives further credit for the idea that changes in the alpha rhythm are related to mindfulness training, however, up to now there seems to be no evidence that changes in the alpha rhythm are related to an increased ability to filter out sensory input.

An aspect that seems quite relevant when dealing with painful stimuli like those employed in the current study is gender differences. In our study, the majority of participants was female and previous studies [37,38] revealed that female participants display greater sensitivity to painful stimuli, which may be related to differences in body size and menstrual cycle. Thus, group differences observed in our study could be modulated by gender differences, which is a factor that should be taken into account in future studies with painful stimuli.

## Conclusions

Self-reports on experienced mindfulness indicate that MBSR training has an influence on the experience of our participants. No support was obtained for the view that MBSR affects attentional orienting. One trend effect, a reduction in the P340 in the first session in the post-post2 group seemed in line with our hypothesis, but the presence of the same pattern in the second session does not allow a straightforward conclusion. Examination of the influence of individual training time showed that this factor may have modulated group-specific effects. For example, experienced mindfulness was related to individual training time, and modulated several observed effects, although no influence of this factor was observed on our electrophysiological index of somatosensory spatial attention. However, other observed effects of spatial attention, like the observed differences between sustained and transient spatial attention in the orienting phase, and effects of spatial attention on the N130, N180 and the P340 components corroborated our previous findings. In short, spatial attention has major effects on the orienting towards and the processing of intracutaneous electrical stimuli, but no clear support was obtained that MBSR training modulates these effects.

## Supporting information

S1 Table *F*-values (with *F*(1,26)) for the effect of Attention Condition on lateralized power spectra (LPS) in the α_1_ band for central (C4/3), lateral central (C6/5), centro-parietal (CP4/3), lateral centro-parietal (CP6/5), parietal (P4/3), lateral parietal (P6/5), occipito-parietal (PO4/3), and lateral occipital sites (PO8/7). (PDF)

S2 Table *F*-values (with *F*(1,26)) for deviations from zero (the intercept) for lateralized power spectra (LPS) in the α_1_ band for central (C4/3), lateral central (C6/5), centro-parietal (CP4/3), lateral centro-parietal (CP6/5), parietal (P4/3), lateral parietal (P6/5), occipito-parietal (PO4/3), and lateral occipital sites (PO8/7). Effects demonstrate a contralateral reduction in power.
(PDF)

S3 Table *F*-values (with *F*(1,26)) for the effect of Attention Condition on lateralized power spectra (LPS) in the α_2_ band for central (C4/3), lateral central (C6/5), centro-parietal (CP4/3), lateral centro-parietal (CP6/5), parietal (P4/3), lateral parietal (P6/5), occipito-parietal (PO4/3), and lateral occipital sites (PO8/7). (PDF)

S4 Table *F*-values (with *F*(1,26)) for deviations from zero (the intercept) for lateralized power spectra (LPS) in the α_2_ band for central (C4/3), lateral central (C6/5), centro-parietal (CP4/3), lateral centro-parietal (CP6/5), parietal (P4/3), lateral parietal (P6/5), occipito-parietal (PO4/3), and lateral occipital sites (PO8/7). Effects demonstrate a contralateral reduction in power.
(PDF)
