# Supplementary material for: Does mindfulness training modulate the influence of spatial attention on the processing of intracutaneous electrical stimuli?
Source: PLoS One. 2018 Aug 9;13(8):e0201689. doi: 10.1371/journal.pone.0201689 (PMC6084927; doi:10.1371/journal.pone.0201689)
Supplement: S1 Table — (PDF) [file pone.0201689.s001.pdf]

**Time window**	**C4/3**	**C6/5**	**CP4/3**	**CP6/5**	**P4/3**	**P6/5**	**PO4/3**	**PO8/7**
400-500 ms	20.7***	**46.5*****	37.6***	1.6	0.5	4.8	17.1***	29.3***
500-600 ms	8.0	**151.6*****	2.7	37.0***	5.1	0.0	12.7*	20.0***
600-700 ms	7.1	**23.8*****	1.6	0.5	0.4	4.8	8.5	7.5
700-800 ms	0.3	0.3	52.0***	0.1	**66.2*****	26.1***	49.1***	0.7
800-900 ms	28.0***	92.6***	**125.0*****	60.7***	85.8***	69.2***	44.7***	18.2***
900-1000 ms	**74.0*****	67.8***	38.6***	19.1***	5.6	1.2	3.1	1.0

* $p < 0.005$, ** $p < 0.001$, *** $p < 0.0005$. Highest *F*-values per time window are indicated in bold.
