# Supplementary material for: Does mindfulness training modulate the influence of spatial attention on the processing of intracutaneous electrical stimuli?
Source: PLoS One. 2018 Aug 9;13(8):e0201689. doi: 10.1371/journal.pone.0201689 (PMC6084927; doi:10.1371/journal.pone.0201689)
Supplement: S2 Table — Effects demonstrate a contralateral reduction in power. (PDF) [file pone.0201689.s002.pdf]

Time window	C4/3	C6/5	CP4/3	CP6/5	P4/3	P6/5	PO4/3	PO8/7
400-500 ms	**22.6*****	0.2	0.4	2.5	3.4	13.7*	3.5	17.8***
500-600 ms	3.2	7.1	0.1	0.5	0.0	**10.1***	6.7	8.3
600-700 ms	5.7	0.0	12.0*	**21.8*****	8.0	0.7	5.0	7.1
700-800 ms	4.4	7.8	28.0***	**44.5*****	5.3	4.6	0.0	1.0
800-900 ms	8.4	2.0	14.1*	15.2**	0.0	**20.3*****	2.9	17.4***
900-1000 ms	7.5	2.8	13.2*	15.8***	0.0	**15.8*****	3.8	13.7*

* $p < 0.005$, ** $p < 0.001$, *** $p < 0.0005$. Highest F-values per time window are indicated in bold.
