# Supplementary material for: Does mindfulness training modulate the influence of spatial attention on the processing of intracutaneous electrical stimuli?
Source: PLoS One. 2018 Aug 9;13(8):e0201689. doi: 10.1371/journal.pone.0201689 (PMC6084927; doi:10.1371/journal.pone.0201689)
Supplement: S3 Table — (PDF) [file pone.0201689.s003.pdf]

**Time window**	**C4/3**	**C6/5**	**CP4/3**	**CP6/5**	**P4/3**	**P6/5**	**PO4/3**	**PO8/7**
400-500 ms	2.9	50.8***	13.3*	17.5***	0.0	16.4***	2.7	**55.0*****
500-600 ms	16.7***	29.0***	28.7***	**236.6*****	15.2**	97.5***	48.3***	0.0
600-700 ms	65.3***	6.0	14.8**	85.2***	4.3	**231.1*****	36.4***	8.3
700-800 ms	10.7*	28.6***	2.9	19.4***	2.0	**91.5*****	30.9***	28.8***
800-900 ms	0.0	51.2***	34.9***	55.6***	**85.1*****	64.9***	56.1***	31.6***
900-1000 ms	15.2**	37.6***	98.8***	**192.0*****	143.1***	118.2***	102.7***	123.4***

* $p < 0.005$, ** $p < 0.001$, *** $p < 0.0005$. Highest *F*-values per time window are indicated in bold.
