# Supplementary material for: Does mindfulness training modulate the influence of spatial attention on the processing of intracutaneous electrical stimuli?
Source: PLoS One. 2018 Aug 9;13(8):e0201689. doi: 10.1371/journal.pone.0201689 (PMC6084927; doi:10.1371/journal.pone.0201689)
Supplement: S4 Table — Effects demonstrate a contralateral reduction in power. (PDF) [file pone.0201689.s004.pdf]

**Time window**	**C4/3**	**C6/5**	**CP4/3**	**CP6/5**	**P4/3**	**P6/5**	**PO4/3**	**PO8/7**
400-500 ms	1.1	1.0	4.2	8.1	0.0	7.0	1.7	2.3
500-600 ms	3.7	2.5	**15.6****	4.0	0.8	8.0	0.1	0.7
600-700 ms	15.3**	0.2	**34.1*****	24.9***	4.6	4.7	0.0	0.0
700-800 ms	29.2***	4.2	38.4***	**42.2*****	1.2	13.2*	4.0	16.5***
800-900 ms	24.5***	1.9	7.8	2.0	16.9***	93.6***	48.3***	**113.6*****
900-1000 ms	18.6***	11.1*	4.4	3.0	23.5***	105.2***	55.3***	**112.5*****

* $p < 0.005$, ** $p < 0.001$, *** $p < 0.0005$. Highest *F*-values per time window are indicated in bold.
